# Enhancing the Phytochemicals and Antioxidant Abilities of Isoflavone-Enriched Soybean Leaves Through Inoculation with *Lacticaseibacillus paracasei* LAB47 and *Levilactobacillus brevis* WCP02

**DOI:** 10.3390/foods14061008

**Published:** 2025-03-16

**Authors:** Hee-Yul Lee, Ji-Ho Lee, Du-Yong Cho, Jong-Bin Jeong, Ga-Yong Lee, Mu-Yeun Jang, Jin-Hwan Lee, Kye-Man Cho

**Affiliations:** 1 Gyeongnam Anti-Aging Research Institute, Sancheong 52215, Republic of Korea; hylee0614@gari.or.kr; 2 Department of Green Bio Science and Agri-Food Bio Convergence Institute, Gyeongsang National University, Jinju 52725, Republic of Korea; 3 Department of Smart Green Resources, Dong-A University, 37, Nakdong-daero 550 beon-gil, Saha-gu, Busan 49315, Republic of Korea

**Keywords:** isoflavone-enriched soybean leaves, lactic acid bacteria, γ-aminobutyric acid, isoflavone, digestive enzyme inhibitory, DNA damage protection activity

## Abstract

In this study, *Lacticaseibacillus paracasei* LAB47 (LAB47) and *Levilactobacillus brevis* WCP02 (WCP02) were selected for the fermentation of isoflavone-enriched soybean leaves (IESLs) according to their survival capability under artificial gastric acid, β-glucosidase activity, γ-aminobutyric acid (GABA) production ability, and isoflavone contents. The strain ratio and fermentation duration with LAB47 and WCP02 for IESLs were 1:1 (per 2.5%, *v*/*v*) with a fermentation time of 72 h. Finally, the fermented IESLs (FIESLs) were compared with the raw and steamed IESLs (RIESLs and SIESLs, respectively) to determine the fatty acids, free amino acids, isoflavones, antioxidant activities, digestive inhibitory activities, and DNA protection capacity. The contents of total fatty acids (1295.67 mg/100 g), GABAs (101.39 mg/100 g), total phenolics (33.73 gallic acid equivalents mg/g), total flavonoids (13.93 rutin equivalents mg/g), and isoflavone aglycones (2588.85 μg/g) were higher in FIESLs than in RIESLs and SIESLs. In addition, the IC_50_ inhibition of glucosidase (2.85 mg/mL) and pancreatic lipase (4.38 mg/mL) and DNA damage protection activities were superior in FIESLs than in RIESLs and SIESLs. Therefore, FIESLs with LAB47 and WCP02 increased the phytochemical and antioxidant activities of IESLs and may be used as functional foods.

## 1. Introduction

Soybean leaves are by-products of soybeans and are not widely used. In South Korea, they are mainly consumed in Gyeongsangnam-do and Jeju Island as pickles or wraps. However, interest in soybean leaves is increasing because they were reported to also contain key functional compounds, such as isoflavones. In particular, Yuk et al. [1] were the first to report an increase in isoflavones when ethylene, a plant growth hormone, was applied at a specific stage during cultivation. In addition, studies using isoflavone-enriched soybean leaves (IESL) are progressing gradually. Lee et al. [2] confirmed an increase in the antioxidant activities, γ-aminobutyric acid (GABA), and total phenolic and flavonoid contents through the solid lactic acid fermentation of IESL.

Fermentation is important in our daily lives and is integral to many cultures, each with their signature fermented foods. Various microorganisms, including fungi and lactic acid bacteria (LAB), are commonly used in fermentation. Studies have shown that LAB fermentation can transform substrates by converting compounds, such as linolenic acid, glutamic acid (GA), and isoflavone glycoside, into more bioactive forms, such as conjugated linoleic acid, GABA, and isoflavone aglycone [2,3]. Notably, GABA serves as the primary inhibitory neurotransmitter in the brain and aglycones produced through fermentation are more easily digested and absorbed by the body, with greater bioavailability than glycosides [4,5,6].

The transformation of GA into GABA is facilitated by glutamate decarboxylase (GAD), with pyridoxal-5′-phosphate as a necessary cofactor for the reaction [7]. This conversion of isoflavone glycosides to aglycones is deglycosylated by β-glycosidases [8]. Various LAB strains can produce β-glycosidases and GAD. However, the abilities of these LAB strains to produce GABA and aglycones vary, leading to different production levels [7,8]. Furthermore, a study showed that combining multiple LAB strains during fermentation greatly enhanced functional components compared with single-strain fermentation [3].

Therefore, this study examined the fermentation characteristics of IESL by different LAB strains and suitable strains for IESL fermentation were selected. Ultimately, the most suitable strain, strain ratio, and fermentation duration for IESL were determined and the differences between raw and steamed IESL (RIESLs and SIESLs, respectively) were compared and analyzed.

## 2. Materials and Methods

### 2.1. Preparation of IESL

In this study, the soybean (*Glycine max* (L.) Merrill) seeds (yellow soybean: variety *Yutae*) were purchased from the *Seohyun* Pharmaceutical Farming Association (Nonsan, Chungcheongnam-do, South Korea). In order to harvest the IESL, soybean seeds (*Yutae* seeds) were sown in pots, cultivation in the plant factory cube was maintained at 23 ± 2.0 °C, 50 ± 10% relative humidity, and 180 ± 10 µmol· m^−2^·s^−1^ photosynthetic photon flux density (PPFD) used white, red, and blue LEDs (LH351H, Samsung LED, Suwon, South Korea) over a 16 h photoperiod. The soybean leaves were grown until the pre-flowering stage (V4 stage: third trifoliate fully expanded) at a time with a growth period of about 30 days after planting and the height of the plant was 70 cm on average. PPFD levels were measured in a horizontal plane of 25 cm vertical distance from the light source at five spots (the center of the tray and four corners) using a spectroradiometer (LI-180; Li-Cor, Lincoln, NE, USA). After 30 days, the plants were transferred to a specially designed smart chamber, where they were treated once with 5000 mg/kg of ethylene and harvested after 24 h. The smart chamber maintained the same conditions as the plant factory. After harvest, they were washed, dried, pulverized, and then stored at −40 °C (frozen).

### 2.2. Medium, Reagents, and Instruments

Lactobacilli MRS broth and agar (MRSB/MRSA, Difco, Becton-Dickinson, Sparks, MD, USA) were used as the LAB culture medium. The G-spin Genomic DNA Purification Kit (iNtRON Biotechnology, Suwon, Republic of Korea), PCR Purification Kit (iNtRON Biotechnology, Kirkland, WA, USA), a thermal cycler (Mastercycler Pro S, Eppendorf, Hamburg, Germany), electrophoresis apparatus (Mupid-21, Optima Inc., Tokyo, Japan), and an UV transilluminator (BioDoc-It™ 220 Imaging System, Analytik Jena, Jena, Germany) were used for LAB identification. The GA, GABA, and ninhydrin used for the thin-layer chromatography (TLC) analysis were purchased from Sigma-Aldrich Co. (Saint Louis, MO, USA). Other reagents were purchased from Sigma-Aldrich. Instruments for gas chromatography (GC, Nexis GC-2030, Shimadzu Corp., Kyoto, Japan), automatic amino acid analysis (L-8900, Hitachi High-Technologies Co., Tokyo, Japan), high-performance liquid chromatography (HPLC, Agilent 1200 system, Agilent Technologies Inc., Waldbronn, Germany), and spectrophotometry (UV-1800 240 V, Shimadzu Corp., Kyoto, Japan) were used to measure the content of fatty acids, free amino acids, isoflavones, total phenolic content (TPC), and total flavonoid content (TFC).

### 2.3. Characteristic Screening of LAB Strains Isolated from Kimchi

The 363 LAB strains isolated from kimchi have been published previously [9]. Single colonies were purified and cultured in MRS broth. Each culture of 750 µL was mixed with sterile glycerol (250 µL) and stored at −70 °C. First, the 363 strains were examined to measure β-glucosidase activity and isoflavone conversion ability from glycosides to aglycones [10,11]. Then, 13 LAB strains that demonstrated superior ability were selected. Second, the 13 LAB strains were also examined to assess their survival capability under artificial gastric acid [12], β-glucosidase activity [10], GABA production ability using TLC [9], and isoflavone contents [11] for the fermentation of IESLs. The artificial gastric acid survival capabilities of the LAB strains were determined according to previous methods [12] with slight modification. The artificial gastric acid values were obtained in triplicate to ensure accuracy. First, each LAB strain was cultured for 48 h at 37 °C in MRS broth. The prepared LAB culture was added at 2% (*v*/*v*) to 1% (*v*/*v*) MRS broth at various pH levels (2.0, 2.5, and 3.0) and inoculated for 0, 2, and 4 h at 37 °C. At this time, the pH levels were adjusted using HCl and NaOH and 1% pepsin was added. The number of viable cells after inoculation was counted and the survival rate was calculated in comparison with the viable cell count after 0 h. The β-glucosidase activity, GABA production ability by TLC, and isoflavone contents were determined as described previously [9,10,11] and the measurements were performed in quintuplicate to ensure accuracy, except TLC. Finally, LAB47 was selected and identified based on the *16S rRNA* gene amplified using polymerase chain reaction techniques [13]. The phylogenetic relationships with other microorganisms were analyzed based on the identified 16S rRNA sequences, and a phylogenetic tree was constructed using DNAman software version 4.11 (Lynnon Biosoft, Vaudreuil-Dorion, QC, Canada).

### 2.4. Fermentation Condition Optimization for IESL

The fermentation optimization was determined through comparisons based on the mixed strains, strain ratios, and fermentation times. First, before fermenting IESL, 9.5 g of IESL powder, 0.5 g of sucrose, and 50 mL of distilled water were mixed and sterilized at 121 °C for 30 min. Based on the results of a single fermentation, *Lacticaseibacillus paracasei* LAB47 (LAB47) was selected among the 13 strains. In addition, *Levilactobacillus brevis* WCP02 (WCP02) was used with LAB47 for mixed-strain fermentation, which has an excellent GABA production rate [14]. LAB47 and WCP02 single and mixed fermentations were performed. The sterilized IESL were fermented with LAB47 (5%, *v*/*v*), WCP02 (5%, *v*/*v*), and LAB47 + WCP02 (each 2.5%, *v*/*v*). Second, mixed fermentation was performed for two strains at five different ratios of LAB47:WCP02 (1:1, 1:2, 3:1, 1:2, and 1:3 at 5% (*v*/*v*)). Subsequently, in the selected ratio, fermentations were performed at eight timepoints: 0, 6, 12, 24, 36, 48, 72, and 96 h. After fermentation, all samples were dried (55 °C for 48 h) and ground. Samples were stored at −40 °C until used as needed. The optimization condition was selected according to the measured amino acids (such as GA and GABA) and isoflavone contents. GA and GABA contents were analyzed using a previously published method [2] with modifications. The isoflavone analysis was performed by HPLC according to a previously described method [11]. The bioconversion rate from isoflavone glycoside types (malonylglycosides or glycosides) to aglycone types was calculated using the following Formula (1):

Isoflavone bioconversion rate (%) = (C_aglycone_/C_total_) × 100 (1)

C_aglycone_: isoflavone aglycone type contents; C_total_: total isoflavone contents.

### 2.5. Characteristics of IESL According to the Processing Stage

#### 2.5.1. Processing Conditions of IESL

First, RIESL were dried and ground to a size of 100 mesh. Second, SIESL were prepared as follows: RIESL were mixed with distilled water (five-fold of the IESL weight, *v*/*w*) and sterilized at 121 °C for 30 min. FIESL were prepared using sucrose (2% of the RIESL weight) and tap water (five-fold of the RIESL weight, *v*/*w*) and sterilized at 121 °C for 30 min. The sample was cooled to 35 °C and starter cultures of *L. paracasei* LAB47 and *L. brevis* WCP02 were inoculated at 2.5% and then fermented at 35 °C for 72 h. Then, SIESL and FIESL were dried (for 48 h at 55 °C) and ground (100 mesh size). RIESL, SIESL, and FIESL were stored at −70 °C until use in the experiments.

#### 2.5.2. Analysis of Physicochemical Properties

The pH measurements were performed using a pH meter (Orion STAR A211, Thermo Fisher Scientific, Waltham, MA, USA). Total acidity was measured by neutralization titration, where 1 g of the sample was titrated to pH 8.2 ± 0.1 using 0.1 N NaOH, and the amount of NaOH consumed was measured and converted to lactic acid equivalents. Viable cells were counted by plating serial dilutions of the sample on MRS agar plates and incubated at 30 °C for 48 h and colonies were counted. The measurements were performed in quintuplicate to ensure accuracy.

#### 2.5.3. Fatty Acid Measurement

The fatty acid analysis was performed following the slightly modified method described by Tammekivi et al. [15]. Initially, 25 mg of the sample was combined with 0.5 mL of the internal standard triundecanoin (TAG C_11_ at a concentration of 2 mg/mL) and 1.5 mL of a 0.5 N NaOH methanol solution. This mixture was heated to 100 °C for 5 min to hydrolyze the fats into fatty acids and glycerol. After cooling, 2 mL of the BF3-methanol solution (approximately 14%, Supelco, Bellefonte, PA, USA) was added and mixed thoroughly. The mixture was heated again at 100 °C for 30 min to facilitate methyl esterification. Once cooled, 1 mL of iso-octane was added and mixed vigorously. Following this, 5 mL of the saturated sodium chloride solution was introduced and stirred. After reaching room temperature, only the iso-octane layer was separated, dehydrated using anhydrous sodium sulfate, and filtered through a 0.45-μm membrane filter (Dismic-25CS, Toyoroshikaisha, Ltd., Tokyo, Japan) for GC analysis. An SP-2560 capillary GC column (100 m × 0.25 mm i.d., with 0.2 µm film thickness, Supelco) equipped with a flame ionization detector was used. Then, an injection volume of 1 µL was used, with the injector temperature set at 225 °C, operating in the split mode with a split ratio of 200:1. Helium was used as the carrier gas at a flow rate of 0.75 mL/min. The oven temperature was held at 100 °C for 4 min, increased to 240 °C at a rate of 3 °C/min, and maintained for 15 min. The detector temperature was set to 285 °C. Each fatty acid was identified by comparing it with a standard substance (CRM47885, Supelco 37-component FAME mixture). The measurements were performed in quintuplicate to ensure accuracy.

#### 2.5.4. Measurement of Free Amino Acids

Free amino acids were analyzed following the slightly modified method of Lee et al. [2]. First, 0.1 g of the sample was homogenized with 5 mL of distilled water and then hydrolyzed at 60 °C for 1 h using a heating block. Subsequently, 1 mL of 10% 5-sulfosalicylic acid dihydrate was added, mixed, and then left at 4 °C for 2 h. The mixture was then centrifuged at 15,000 rpm for 3 min and filtered through a syringe filter. The filtrate was concentrated under reduced pressure at 50 °C using a rotary evaporator and then dissolved in 2 mL of pH 2.2 lithium buffer, followed by filtration through a 0.45 μm membrane filter. The final filtered sample was quantitatively analyzed using an automatic amino acid analyzer. The measurements were performed in quintuplicate to ensure accuracy.

#### 2.5.5. Extract Preparation

Each 1 g of the sample was combined with 40 times its volume of 50% methanol (40 mL) and stirred at 3000× *g* for 12 ± 2 h at 20 °C for extraction. Afterward, the mixture was centrifuged at 1000× *g* for 10 min at 20 °C. The resulting supernatant was then filtered through a 0.45-μm membrane filter for the measurement of isoflavones, TPC, TFC, antioxidant activities, and digestive enzyme inhibitory activities.

#### 2.5.6. Measurement of Isoflavones

The isoflavone analysis was performed using HPLC according to the method described by Lee et al. [11]. The analysis was carried out using Lichrophore 100 RP C18 columns (LichroCART 125–4, 5 µm, 125 mm × 4 mm, Merck KGaA, Darmstadt, Germany). Mobile Phases A and B were HPLC water with 0.2% acetic acid (*v*/*v*) and HPLC acetonitrile with 0.2% acetic acid (*v*/*v*), respectively. The conditions for the mobile phase solvents were set based on Solvent B as follows: 0% (0 min), 10% (15 min), 20% (25 min), 25% (30 min), and 35% (45–50 min). The analysis conditions were a sample injection volume of 20 µL, flow rate of 1 mL/min, column temperature of 30 °C, and detection wavelength of 254 nm. A diode array detector was used for the analysis. The measurements were performed in quintuplicate to ensure accuracy. The peak of each substance was identified by comparing it with the retention time of the standard substances and the content was quantified using the calibration curve of each standard substance. Calibration curves were created using seven concentration points (1.0, 0.8, 0.6, 0.4, 0.2, 0.1, and 0.05 mg/mL) derived from a 1 mg/mL stock solution for each standard, with a correlation coefficient (r^2^) > 0.998. The bioconversion rate from isoflavone glycoside types (malonylglycosides or glycosides) to aglycone types was calculated using Formula (1).

#### 2.5.7. Measurement of TPC and TFC

TPC and TFC were measured as previously described [2] and calculated using standard curves with gallic acid and rutin (1, 5, 10, 20, 50, 100, 200, and 500 μg/mL), respectively. In addition, TPC and TFC were expressed as mg/g of gallic acid equivalents (GAEs) and mg/g of rutin equivalents (REs), respectively. The measurements were performed in quintuplicate to ensure accuracy.

#### 2.5.8. Measurement of Antioxidant Activities

The DPPH and ABTS radical scavenging activities were determined using the method published by Belmonte-Herrera et al. [16]. The antioxidant activities were measured in six concentrations and expressed as IC_50_ values (mg/mL), following the method outlined by Adinortey et al. [17]. The measurements were performed in quintuplicate to ensure accuracy.

#### 2.5.9. Measurement of the Digestive Enzyme Inhibitory Activities

The activities of α-glucosidase and lipase inhibition were assessed using a previously described method [18]. For α-glucosidase inhibitory activity, 50 μL of 200 mM sodium phosphate buffer (pH 6.8) was mixed with 70 μL of 1 U/mL α-glucosidase and 30 μL of each sample and then preincubated at 37 °C for 10 min. Moreover, 100 μL of 10 mM *p*-NPG dissolved in 200 mM sodium phosphate buffer (pH 6.8) was added and the mixture was incubated at 37 °C for 10 min. The reaction was stopped by adding 750 μL of 100 mM Na_2_CO_3_ and the absorbance was measured at 420 nm. For the inhibitory activity of pancreatic lipase, the same procedure was followed, except that pancreatic lipase (enzyme) and 10 mM *p*-NPB (substrate) were used. In both assays, the negative control (CTL) used an extraction solvent instead of the sample. Inhibitory activity was assessed at five different concentrations and a logarithmic regression curve was created to determine the IC_50_ values (mg/mL):

Digestive enzyme inhibitory activity (%) = [1 − (A/B)] × 100(2)

A: absorbance of an experimental sample, B: absorbance of the negative control.

#### 2.5.10. Measurement of the DNA Protection Capacity

The ability to protect supercoiled plasmid DNA (pBluescript SK(+) Easy from *E. coli*) from hydroxyl radicals was evaluated following the slightly modified methods of Adinortey et al. [17] and Lee et al. [19]. Plasmid DNA was isolated using 1 × TE buffer (10 mM Tris-Cl and 1 mM EDTA = elution buffer) and Fenton’s reagent was prepared by mixing 100 mM H_2_O_2_, 0.1 mM acetic acid, and 1.6 mM FeCl_3_. The reaction mixture (20 μL) containing 2 μL of plasmid DNA, 10 μL of the sample, and 8 μL of Fenton’s reagent was incubated at 30 °C for 1 h. After incubation, the DNA gel loading dye (6×) was added to the reaction mixture and mixed well and 10 μL of the final mixture was loaded onto a 1.2% agarose gel. The gel was subjected to electrophoresis at 50 V for 80 min to visualize DNA, which was then photographed using an UV transilluminator.

### 2.6. Statistical Analysis and Data Processing

The results of all experiments are expressed as mean ± SD. Analysis of variance (ANOVA) was performed using SAS 9.4 (SAS Institute, Cary, NC, USA). Tukey’s multiple-comparison tests were performed at the level of *p* < 0.05 to determine the significance of the ANOVA results. Data processing and graphing were performed using Microsoft Excel (Microsoft 365, Microsoft Corporation, Redmond, WA, USA). The heatmap and principal component analysis (PCA) data were analyzed and visualized for correlations using MetaboAnalyst 6.0 (http://www.metaboanalyst.ca) (accessed on 5 February 2024).

## 3. Results and Discussion

### 3.1. Selection of LAB47 as the Optimal Fermentation Strain

#### 3.1.1. Artificial Gastric Acidic Tolerance of the 13 LAB Strains

The probiotic potential of the 13 isolated LAB strains was evaluated by assessing their tolerance to artificial gastric acid. The results of the artificial gastric acidic tolerance test are shown in Table 1. At pH 3.0, LAB10, LAB12, LAB20, and LAB48 survived for 2 h but succumbed after 4 h, whereas the remaining strains survived up to 4 h. At pH 2.5, after 2 and 4 h, LAB07 (64.05% and 26.39%), LAB11 (63.32% and 28.57%), LAB21 (46.34% and 7.36%), LAB47 (62.22% and 24.75%), and LAB49 (54.85% and 19.16%) showed high survival rates, whereas other strains either succumbed or exhibited low survival rates. At pH 2.0, most strains succumbed; however, after 2 and 4 h, LAB07 (24.72% and 6.26%), LAB11 (29.05% and 9.25%), and LAB47 (20.63% and 4.61%) showed relatively higher survival rates.

Probiotics are beneficial microorganisms essential for human gut health and enhancing the immune system [20]. In this study, the acid, artificial gastric acidic, and bile acid tolerance capabilities of 13 LAB strains were evaluated. These evaluations are crucial to determining the ability of probiotics to survive passage through the digestive system. In particular, LAB07, LAB11, LAB47, and LAB49 demonstrated overall superior survival rates in all tolerance tests. This finding indicates that these strains may help maintain intestinal microbial balance, promote digestive health, and enhance the immune system. Therefore, these strains are anticipated to play important roles in dietary supplements, functional foods, etc.

#### 3.1.2. β-Glucosidase Activity of the 13 LAB Strains

The β-glucosidase activity of the 13 isolated LAB strains was evaluated and isoflavones in IESL fermentation using the 13 isolated LAB strains were determined (Figure S1). The strains with high β-glucosidase activity were LAB47 (24.3 unit/g) and LAB02 (22.4 unit/g). In addition, the β-glucosidase activities of the LAB10 and LAB12 were comparable at 17.8 and 18.2 units/g; LAB 20, 21, 22, 39, 44, and 48 were not significantly different. Conversely, the β-glucosidase activities of LAB07 (16.2 unit/g), LAB11 (15.8 unit/g), and LAB49 (16.4 unit/g) were lower than those of the other strains (Figure S1).

Hydrolytic enzymes can break down phenolic compounds bound to plant cell walls and hydrolyze bonds, such as glycosides, to release and generate free phenolic compounds and active phenolic forms [21,22]. Among hydrolases, β-glucosidase hydrolyzes glycosidic bonds to release glucose and can convert the glycosidic types of phenolic compounds into active phenols. According to [23], increasing β-glucosidase activity changes the phytochemicals in the fermentation product during fermentation. In particular, major isoflavones (daidzin, malonyl daidzin, genistin, and malonyl genistin) in soybeans are converted to aglycones [23,24]. The isoflavone contents of IESL exist in glycoside types through glycosidic bonds. Therefore, LAB47, which exhibits high β-glucosidase activity, shows the potential to convert them into aglycone types.

#### 3.1.3. GABA Production Ability of LAB

To confirm the GABA production ability of the 13 isolated LAB strains, TLC was performed, as shown in Figure S2. All strains exhibited a band at the same position as GA; however, no bands were observed for GABA, indicating that none of the strains significantly produced GABA.

GA can be converted to GABA by the action of GAD produced by LAB. In the confirmation of GABA production by TLC, the 13 isolated LAB strains showed a pattern identical to that of 5% GA, indicating the absence of GAD activity. Hwang et al. [25] confirmed that *L. brevis* can produce GABA. *L. brevis* WCP02, known for its excellent probiotic properties and GABA production, was used for mixed fermentation to enhance GABA production.

#### 3.1.4. Isoflavone Conversion Rate of LAB

**Figure 1:**
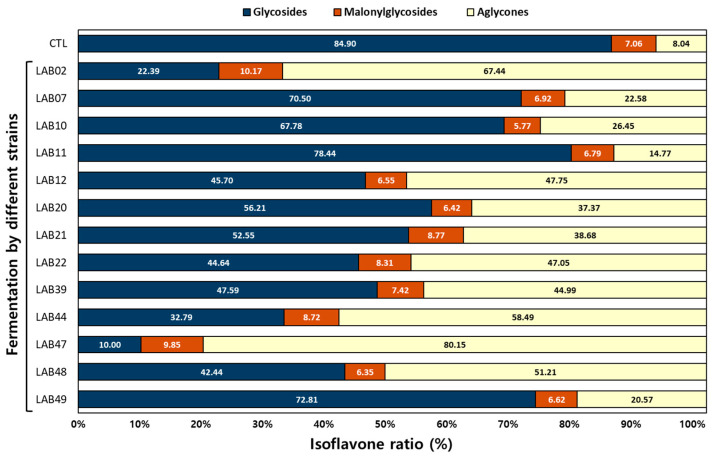
Comparison of isoflavone ratio of isoflavone in IESL fermentation using different lactic acid bacteria. Fermentation conditions: The isolavone-enriched soybean leaves were fermented at 30 °C for 120 h using 13 isolated lactic acid bacterial strains. All values are presented as the mean ± SD of pentaplicate determinations and different small letters correspond to the significant differences relating to the fermented lactic acid bacterial strains using Tukey’s multiple test (*p* < 0.05).

The isoflavone conversion rate in fermented IESL was examined using 13 isolated LAB strains (Figure 1 and Table S1).

To optimize the IESL fermentation conditions, single fermentations were performed using 13 strains and changes in the isoflavone content in the fermentation products were analyzed. The isoflavone conversion rate and content are shown in Figure 1 and Table S1; compared with the CTL, the peak levels of isoflavone malonylglycosides, glycoside types, and aglycone types indicate that LAB11 exhibited a low conversion rate to aglycones, whereas LAB47 and LAB02 had higher conversion rates. When comparing the contents shown in Table S1, the isoflavone contents in glycosides (malonylglycosides and glycosides) in the CTL, LAB02, LAB11, and LAB47 were 6489.01, 1356.43, 5439.75, and 792.97 μg/g, respectively. Furthermore, the isoflavone contents in aglycone types were 597.29, 2809.92, 942.86, and 3202.04 μg/g, respectively. The isoflavone aglycone rates among the detected total isoflavones were 8.43, 67.44, 14.77, and 80.15%, respectively. As shown in Table S1, significant differences were noted in the isoflavone content between strains.

LAB possess different metabolic characteristics depending on the species and are used for various purposes, such as enhancing the flavor of fermented foods, increasing nutritional value, reducing harmful substances, and extending shelf life [26]. Hwang et al. [14] demonstrated that the fermentation of soy milk with LAB can result in the bioconversion of isoflavones due to the action of β-glucosidase. In the present study, each strain exhibited different enzyme activities, resulting in various isoflavone bioconversion patterns. Therefore, mixed fermentations were performed using LAB47, the strain with the highest conversion rate to aglycones.

#### 3.1.5. Identification of LAB47 Based on 16S rRNA Gene Sequencing

**Figure 2:**
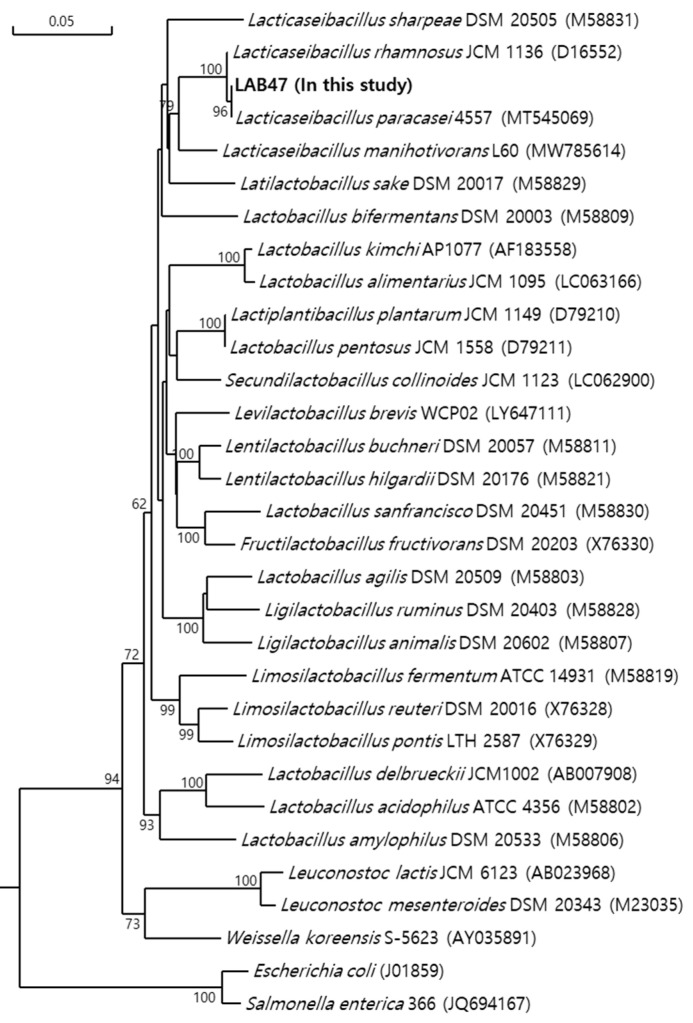
Phylogenetic tree based on 16S rRNA gene sequences of different lactic acid bacteria. Numbers above each node are confidence levels (%) generated from 1000 bootstrap trees. The scale bar is in fixed nucleotide substitutions per sequence position.

Phylogenetic analysis was performed to determine taxonomic positions and the results indicated the classification of *L. paracasei* strains, which formed similar phylogenetic groups within each genus (Figure 2 and Figure S3).

### 3.2. Fermentation Condition Optimization for IESL

#### 3.2.1. Analysis of GA and GABA Contents According to Fermentation Conditions

**Figure 3:**
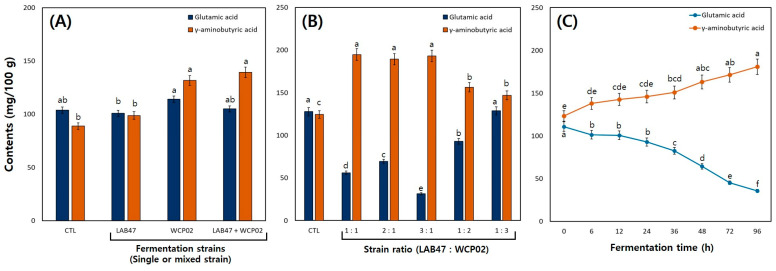
Comparison of glutamic acid and γ-aminobutyric acid contents in IESL according to fermentation lactic acid bacteria strains (single or mixed), strain ratio, and fermentation time. (**A**) single or mixed strains, (**B**) strain ratio, and (**C**) fermentation time. Fermentation conditions: (**A**) the IESL was fermented at 30 °C for 120 h with single and mixed strains of LAB47 and WCP02, (**B**) the IESL was fermented at 30 °C for 120 h with the mixing ratio of LAB17 and WCP02 strains, and (**C**) the IESL was fermented at 30 °C for 96 h using the cocktail of LAB47 and WCP02 strains during fermentation. All values are presented as the mean ± SD of pentaplicate determinations and different small letters correspond to the significant differences relating to the fermented lactic acid bacterial strains using Tukey’s multiple test (*p* < 0.05).

To optimize the IESL fermentation conditions, mixed fermentation was performed using LAB47, which showed excellent conversion to isoflavone aglycones, and *L. brevis* WCP02, recognized for its good probiotic properties and GABA production. The difference in the GA and GABA contents in the single and mixed fermentation samples is shown in Figure 3A. The GA contents were 103.86, 100.84, 114.16, and 105.00 mg/100 g in the CTL, LAB47, WCP02, and LAB47 + WCP02, whereas the GABA contents were 88.80, 98.80, 131.63, and 139.35 mg/100 g, respectively (Figure 3A). The GABA content of LAB47 was lower than that of WCP02; however, the highest GABA content was observed when LAB47 and WCP02 were fermented together (Figure 3A).

The TLC results indicated that LAB47 did not produce GABA. However, as Hwang et al. [25] confirmed the GABA production ability of WCP02, it was additionally used in mixed fermentation, which explained the difference in GABA production between the two strains. In particular, GABA production increased when the two strains were cofermented. Lee et al. [2] supported these findings as GABA production increased when soybean leaves were fermented with a mixture of *Lactiplantibacillus plantarum* P1201 and *Levilactobacillus brevis* BMK184. Therefore, to optimize the IESL fermentation conditions, fermentations were performed with varying ratios using LAB47 and WCP02.

The GA and GABA contents of the fermented products according to the LAB47 and WCP02 mixing ratios are shown in Figure 3B. For the CTL and LAB47:WCP02 in ratios of 1:1, 2:1, 3:1, 1:2, and 1:3, the GA contents were 127.94, 56.15, 69.52, 31.44, 93.04, and 128.79 mg/100 g and the GABA contents were 124.51, 194.84, 189.39, 193.10, 156.43, and 146.97 mg/100 g, respectively (Figure 3B). The GABA content was higher in all mixing ratios than that in the CTL. Notably, when the proportion of LAB47 was equal to or higher than that of WCP02, the GABA content was also higher.

In this study, mixed fermentations were performed by adding WCP02 because of the low GABA production capacity of LAB47. Interestingly, the GABA content increased more significantly when the proportion of LAB47 was higher in the mixing ratio of the two strains. In the study by Lee et al. [2], although *L. plantarum* P1201 had lower GABA production rates than *L. brevis* BMK184 during mixed fermentation, the pH reduction caused by P1201 could influence GABA production in mixed fermentation. Furthermore, Cui et al. [27] reported the varying genetic characteristics involved in the GABA production system among different LAB species. Considering the fermentation conditions and pH of the fermented products, this difference could be a synergistic effect of the genetic characteristics of LAB47 and WCP02. Therefore, the most optimal GABA production was observed at a LAB47:WCP02 mixing ratio of 1:1. Finally, fermentations were performed in the LAB47:WCP02 mixing ratio of 1:1 with varying fermentation times. Changes in the GA and GABA contents according to the fermentation times can be confirmed in Figure 3C. The GABA contents at 0, 12, 24, 72, and 96 h were 123.35, 142.68, 146.11, 171.37, and 180.91 mg/100 g, respectively. The highest GABA content was observed at 96 h; however, the conversion rate from GA to GABA, calculated over the fermentation time, showed a continuous increase from 6 h to 72 h, at 52.65%, 57.66%, 58.60%, 61.14%, 64.63%, 71.68%, 79.14%, and 83.50% but decreased from 96 h onward (Figure 3C). As shown in Figure 3C, GA and GABA contents increased or decreased in inverse proportion to the fermentation time.

In the study by Lee et al. [2], the GAD activity of the fermentation strains increased up to 48 h and then decreased from 72 h onward. Similarly, in the present study, the GABA content continued to increase, whereas the bioconversion rate decreased, which could be attributed to the reduction in GAD activity. Therefore, considering the changes in the GA and GABA contents over the fermentation time, a 72 h fermentation period is the most efficient for GABA production.

#### 3.2.2. Analysis of Isoflavone Contents According to Fermentation Conditions

**Figure 4:**
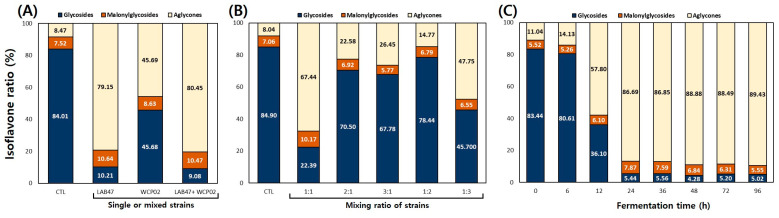
Comparison of isoflavone rates in IESL according to fermentation lactic acid bacteria strains (single or mixed), strain ratio, and fermentation time. (**A**) single or mixed strains, (**B**) strain ratio, and (**C**) fermentation time. Fermentation conditions: (**A**) the IESL was fermented at 30 °C for 120 h with single and mixed strains of LAB47 and WCP02, (**B**) the IESL was fermented at 30 °C for 120 h with the mixing ratio of LAB17 and WCP02 strains, and (**C**) the IESL was fermented at 30 °C for 96 h using the cocktail LAB47 and WCP02 strains during fermentation. All values are presented as the mean ± SD of pentaplicate determinations.

To optimize the IESL fermentation conditions, changes in the isoflavone contents were analyzed in single and mixed fermentations with LAB47 and WCP02 (Table S2 and Figure 4A). The isoflavone contents in glycoside types (malonylglycosides and glycosides) for CTL, LAB47, WCP02, and LAB47 + WCP02 were 10990.31, 1406.38, 4618.39, and 1279.18 μg/g, respectively, whereas the isoflavone contents in the aglycone types were 1017.29, 5340.25, 3886.01, and 5263.71 μg/g, respectively (Table S2). The isoflavone aglycone rates among the detected total isoflavones were 8.47%, 79.15%, 45.69%, and 80.45% (Figure 4A). As shown in Figure 4A, isoflavone aglycone conversion occurred in all fermented products compared with that in the CTL, with the lowest conversion observed in WCP02.

The difference in aglycone conversion rates between LAB47 and WCP02 can be attributed to the distinct metabolic characteristics and enzyme activities of each strain, as observed in their single fermentations. Interestingly, a high conversion rate was observed even in mixed fermentations with strains showing high and low conversion rates to aglycones. Previous studies demonstrated high conversion rates to isoflavone aglycones in mixed lactic acid fermentation using soy milk and soybean leaves [2,28]. In this study, the mixed fermentation of LAB47 and WCP02 also demonstrated the highest conversion rates. Therefore, considering GABA production and the conversion rate to isoflavone aglycones, the mixed fermentation of LAB47 and WCP02 was adopted and fermentations were carried out with varying ratios of the two strains.

To optimize the IESL fermentation conditions, fermentations were performed with varying ratios of fermentation strains and changes in the isoflavone contents in the fermented products were analyzed according to the mixing ratios (Table S3 and Figure 4B). Isoflavone contents in glycoside types (malonylglycosides and glycosides) for the CTL and LAB47:WCP02 in ratios of 1:1, 2:1, 3:1, 1:2, and 1:3 were 6288.63, 543.57, 589.49, 561.16, 572.52, and 542.78 μg/g, respectively. The isoflavone contents in the aglycone types for the same ratios were 590.38, 3063.31, 3136.08, 2938.90, 3239.27, and 3160.62 μg/g, respectively. The calculated isoflavone rates were 8.58%, 84.93%, 84.18%, 83.97%, 84.98%, and 85.34%, respectively (Figure 4B).

As shown in Figure 4B, conversion to isoflavone aglycones was well achieved in all fermented products compared with that in the CTL. The conversion to isoflavone aglycones was well achieved at all mixing ratios, indicating that the influence of LAB47 was significant and that WCP02 did not inhibit the action of LAB47. Therefore, while the conversion rate to isoflavone aglycones was the highest at the LAB47:WCP02 mixing ratio of 1:3, considering both the GABA production and bioconversion rate to isoflavone aglycones, a 1:1 mixing ratio of LAB47 and WCP02 was adopted for fermentation. The fermentation was then performed at varying fermentation times.

To optimize the IESL fermentation conditions, fermentations were performed in the selected mixing ratio at varying fermentation times and changes in the isoflavone contents were analyzed (Table S4 and Figure 4C). The isoflavone contents in the glycoside types (malonylglycosides and glycosides) at 0, 6, 12, 24, 36, 48, 72, and 96 h were 9320.96, 9049.28, 3094.98, 849.68, 799.09, 698.79, 674.60, and 685.55 μg/g, respectively. Meanwhile, the isoflavone contents in the aglycone types were 1156.47, 1489.48, 4239.78, 5533.05, 5278.02, 5585.99, 5187.58, and 5797.43 μg/g (Table S4) and the calculated isoflavone rates were 11.04%, 14.13%, 57.80%, 86.69%, 86.85%, 88.88%, 88.49%, and 89.43%, respectively (Figure 4C). As shown in Figure 4C, at 12 h of fermentation, conversion to aglycones began, most isoflavones were converted at 24 h, and no significant changes occurred afterward.

The conversion rate to isoflavone aglycones was excellent after 24 h of fermentation. In the fermentation of soybean leaves by Lee et al. [2], the conversion to isoflavone aglycones also increased with the fermentation time; however, the activity of β-glucosidase increased up to 48 h and then decreased at 72 h. Considering these factors, the ideal fermentation time is 72 h.

### 3.3. Characteristics of IESL According to the Processing Stage

#### 3.3.1. Phytochemical Properties of IESL According to the Processing Stage

The physicochemical properties of the IESL at the processing stage are presented in Table 2. The pH values of RIESL and SIESL were 6.19 and 6.17, respectively, which were not significantly different, whereas FIESL showed a slight increase to 6.25 after fermentation. The acidity in FIESL increased proportionally to the pH, measured at 2.07%, 2.07%, and 2.16%, respectively. The viable cell count in FIESL was 8.95 log cfu/g.

In general, LAB tend to produce lactic acid during fermentation, resulting in a decrease in the pH. However, in this study, the pH increased. This could be attributed to the characteristics of the fermenting strains used (LAB47 and WCP02) and fermentation conditions or substrate properties. In the strain selection experiment, when IESL were fermented with each of the 13 LAB strains, the pH values both increased and decreased. In mixed-strain fermentation, the pH values of the fermentation products using LAB47 and WCP02 were not significantly different from that of the CTL. In the study by Hwang et al. [25], the fermentation of soy milk using WCP02 resulted in a decrease in the pH. Therefore, the pH changes observed in the present study are attributable to the characteristics of the microorganisms and substrates involved. Kuerban et al. [28] reported similar findings where the pH increased after lactobacilli fermentation under specific conditions. Furthermore, the high viable cell count indicates a vigorous proliferation of LAB during fermentation, suggesting the appropriateness of the fermentation conditions.

#### 3.3.2. Fatty Acid Contents of IESL According to the Processing Stage

**Figure 5:**
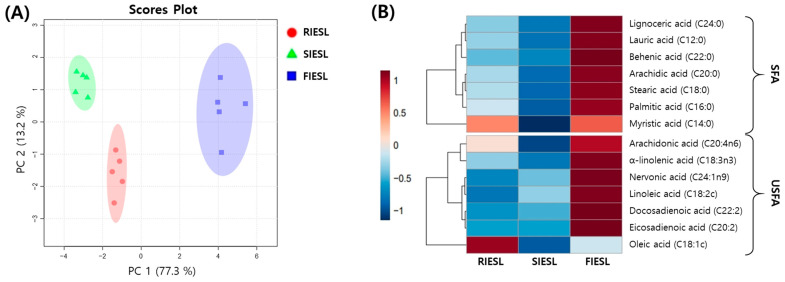
Comparison of fatty acids in isoflavone-enriched soybean leaves during food processing stages. (**A**) score plot of principal component analysis and (**B**) correlation heatmap analysis. Sample: RIESLs, raw isoflavone-enriched soybean leaves; SIESLs, steam isoflavone-enriched soybean leaves; and FIESLs, fermented isoflavone-enriched soybean leaves. SFAs, saturated fatty acids; USFAs, unsaturated fatty acids. Values of the various conditions were normalized and clustered in the heatmap. The color displays the intensity of the normalized mean values of different parameters. The value of *p* < 0.05 was used to determine a statistically significant difference.

Changes in the fatty acid composition according to the processing method of IESL are shown in Table S5 and Figure 5. The total fatty acid contents of RIESLs, SIESLs, and FIESL were 953.90, 910.90, and 1034.00 mg/100 g, respectively (Table S5). In RIESL, SIESL, and FIESL, the major fatty acids included palmitic acid (434.50, 410.30, and 469.70 mg/100 g) and stearic acid (131.10, 126.20, and 140.30 mg/100 g) among saturated fatty acids and α-linolenic acid (127.30, 124.40, and 136.10 mg/100 g) and linoleic acid (109.00, 111.40, and 118.40 mg/100 g) among unsaturated fatty acids (Table S5). PCA showed that 90.5% of the variability was explained by PC1 and PC2, with distinct changes observed based on the processing methods, without overlapping clusters. Based on PC1, which represented 77.3% of the variability, FIESL were positioned further away from RIESL compared with SIESL. This indicates that fermentation had greater effects on the fatty acid content (Figure 5A). The heatmap analysis showed that all fatty acids decreased during sterilization but increased during fermentation (Figure 5B).

Fatty acids serve as an energy source and are crucial components of cell membrane lipids, which can be broken down into fatty acids by enzymes such as lipases. Fermentation using LAB can also facilitate lipid breakdown, although not all strains are capable of it [29]. Ziarno et al. [30] demonstrated that *L. paracasei* BGP1 and *L. brevis* L342 exhibited lipolytic activity during soy milk fermentation, which may explain the increase in fatty acid contents observed in FIESL in the present study. The decrease observed during sterilization can be attributed to the heat treatment that reduces the chemical stability of fatty acids and promotes oxidation reactions [31]. Among the major fatty acids that increase during fermentation, unlike other saturated fatty acids, stearic acid has a neutral effect on total cholesterol and low-density lipoprotein cholesterol levels in the blood and can positively affect cardiovascular health [32]. Changes in the fatty acid composition of IESL according to the processing methods indicate that lactic acid fermentation may positively affect the fatty acid content of IESL. However, an imbalance in fatty acid intake can lead to obesity, diabetes, inflammation, and other diseases, highlighting the importance of appropriate dietary strategies [33].

#### 3.3.3. Free Amino Acid Contents of IESL According to the Processing Stage

**Figure 6:**
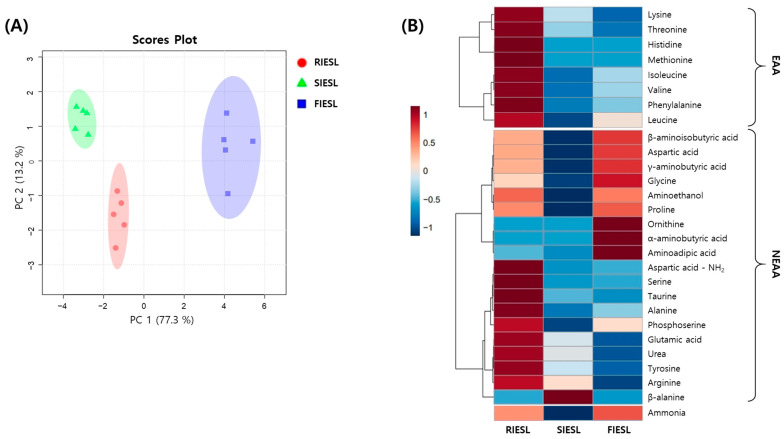
Comparison of free amino acids in isoflavone-enriched soybean leaves during food processing stages. (**A**) score plot of principal component analysis and (**B**) correlation heatmap analysis. Sample: RIESL, raw isoflavone-enriched soybean leaves; SIESL, steam isoflavone-enriched soybean leaves; and FIESL, fermented isoflavone-enriched soybean leaves. EAAs, essential amino acids; NEAAs, non-essential amino acids. Values of the various conditions were normalized and clustered in the heatmap. The color displays the intensity of the normalized mean values of different parameters. The value of *p* < 0.05 was used to determine a statistically significant difference.

Changes in the free amino acid content of IESL according to different processing methods are shown in Table S6 and Figure 6 and Figure S2. The total free amino acid contents were 1899.42, 1221.37, and 1295.67 mg/100 g for RIESL, SIESL, and FIESL, respectively. The major free amino acids included urea (178.73, 116.92, and 64.04 mg/100 g), proline (100.70, 79.65, and 102.82 mg/100 g), aspartic acid (132.01, 98.21, and 141.08 mg/100 g), aspartic acid–NH_2_ (482.73, 328.55, and 339.68 mg/100 g), GA (92.01, 50.57, and 18.21 mg/100 g), alanine (101.22, 67.33, and 75.59 mg/100 g), and GABA (91.53, 61.21, and 101.39 mg/100 g) among non-essential amino acids, as well as valine (95.79, 66.74, and 75.26 mg/100 g) and phenylalanine (92.08, 45.83, and 55.67 mg/100 g) among the essential amino acids (Table S6). The PCA results showed that the variability explained by PC1 and PC2 was 99.2%, indicating distinct changes without overlapping clusters depending on the processing methods (Figure 6A). The heatmap showed a decrease in most of the free amino acids during sterilization, whereas some free amino acids increased during fermentation (Figure 6B).

Amino acids are molecules that exist in free forms, unlike peptides or proteins, in which amino acids are bound together. They can be generated through the action of enzymes, such as proteases or peptidases, and their levels may change during cooking because of interactions with various components under heat. Mesías et al. [34] observed a decrease in amino acid contents during the sterilization of vegetable-based baby food, indicating varied sensitivity to heat among amino acids and resulting in different patterns of change. During fermentation, some amino acids increased, whereas others decreased depending on their types. LAB may require amino acids and peptides as nitrogen sources for their metabolism, which could explain the reduction in free amino acids after fermentation [29]. Similarly, Khan et al. [22] observed a decrease in the total free amino acids during the fermentation of dried longan, and individual amino acids showed a decreasing trend. In addition, an increase in amino acids can occur, similar to an increase in GABA production through the metabolic action of LAB. LAB serve as one of the most important GABA producers, which is synthesized by the decarboxylation reaction catalyzed by GAD [2]. Although GAD characteristics vary among different LAB species and strains, *L. brevis* exhibits a high GABA yield compared with other LAB strains [26]. The GABA content may increase through the lactic acid fermentation of soybeans using *L. brevis*, as demonstrated by Hwang et al. [25]. Consequently, fermented IESL may possess potential functional properties because of the benefits associated with GABA, such as the prevention of hypertension, calming and diuretic effects, reduction in anxiety, and improvement of mood [27].

#### 3.3.4. Isoflavone Contents of IESL According to the Processing Stage

**Figure 7:**
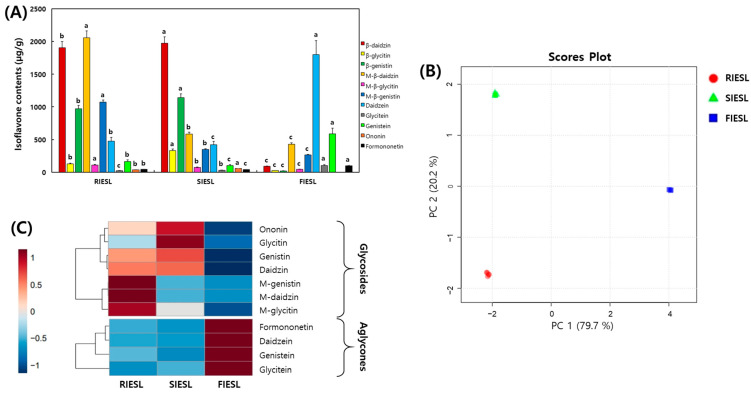
Comparison of isoflavones in isoflavone-enriched soybean leaves during food processing stages. (**A**) isoflavone contents, (**B**) score plot of principal component analysis, and (**C**) correlation heatmap analysis of isoflavones. RIESL, raw isoflavone-enriched soybean leaves; SIESL, steam isoflavone-enriched soybean leaves; and FIESL, fermented isoflavone-enriched soybean leaves. Different small letters on the bar correspond to the significant differences, as determined by Tukey’s multiple-comparison test (*p* < 0.05). The heatmap: the mean values of the various conditions were normalized and clustered in the heatmap. The value of *p* < 0.05 was used to determine a statistically significant difference.

**Figure 8:**
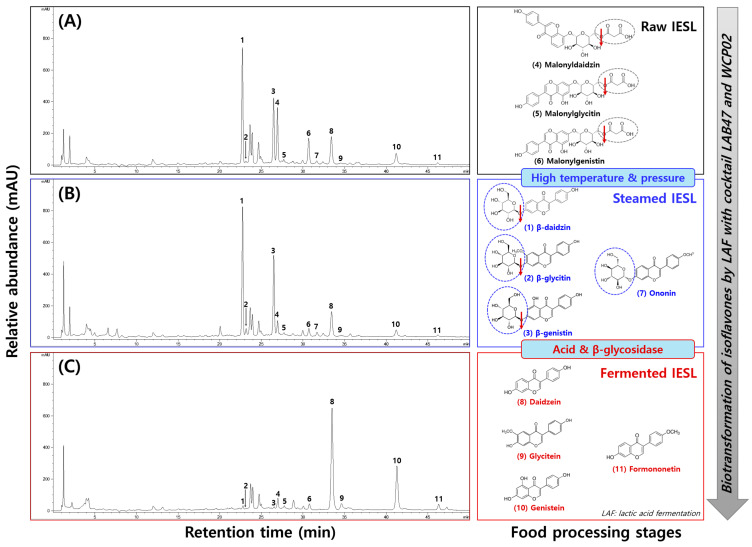
Typical HPLC chromatogram of isoflavones and the bioconversion mechanism of isoflavone compounds of isoflavone-enriched soybean leaves during food processing stages. (**A**) raw isoflavone-enriched soybean leaves (RIESL), (**B**) steam isoflavone-enriched soybean leaves (SIESL), and (**C**) fermented isoflavone-enriched soybean leaves (FIESL). Peak 1, daidzin; Peak 2, glycitin; Peak 3, genistin; Peak 4, malonyldidzin; Peak 5, malonylglycitin; Peak 6, malonylgenistin; Peak 7, ononin; Peak 8, dadzein; Peak 9, glycitein; Peak 10, genistein; Peak 11, formononetin.

Changes in the isoflavone contents of IESL according to different processing methods are shown in Figure 7 and Figure 8. Glycoside types increased slightly before decreasing dramatically to 3041.92, 3502.27, and 135.31 μg/g, respectively. The contents of malonylglycoside types decreased consistently to 3237.67, 1006.28, and 737.12 μg/g.

In contrast, the contents of aglycone types decreased slightly before significantly increasing to 708.21, 593.28, and 2588.85 μg/g, respectively. In particular, the aglycone content in FIESL increased approximately 3.6-fold compared with that in RIESL (Figure 7A). PCA showed that 99.9% of the variability was explained by PC1 and PC2, showing distinct changes without overlapping clusters based on the processing methods (Figure 7B). The heatmaps indicated a decrease in malonylglycoside types and an increase in glycoside types after sterilization, whereas the glycoside types decreased and aglycones types increased after fermentation (Figure 7C). Although the total isoflavone content decreased after the sterilization and fermentation of RIESL, SIESL, and FIESL, the quantities varied between the different types.

In plants, isoflavones typically form glycosidic bonds with sugars and exist in glycoside types. When ingested, they are broken down into aglycones through the enzymatic action of intestinal microorganisms and only a part is absorbed [35]. In particular, malonylglycoside types of isoflavones are presumed to undergo deglycosylation and de-esterification during sterilization [36]. Qu et al. [37] indicated that heating soybeans led to a decrease in malonylglycoside types and an increase in glycoside types. The glycoside types of isoflavones generated by heat can be converted into aglycones by β-glucosidase produced by LAB. Various studies have demonstrated the bioconversion of isoflavones through lactic acid fermentation using different substrates [2,24,25,38]. In this study, the biological conversion of isoflavones occurred depending on the processing method and excellent aglycone conversion rates were demonstrated under the optimal fermentation conditions of IESL. Isoflavones positively affect bone health, cancer prevention, cardiovascular health, antioxidation, and the alleviation of menopausal symptoms. They are absorbed after conversion into aglycone types by enzymes in the intestines, metabolized in the liver into conjugates, and further metabolized by the intestinal microbiota to produce active metabolites, such as *S*-Equol, thus enhancing the physiological activity [39]. The consumption of aglycone type isoflavones after processing would reduce the loss of isoflavones during conversion to aglycones in the intestines, leading to higher absorption rates in the body. Therefore, fermentation using LAB47 and WCP02 offers an effective means of using IESL more efficiently.

#### 3.3.5. TPC and TFC of IESL According to the Processing Stage

Changes in the TPC and TFC of IESLs according to processing methods are shown in Table 3. In general, the contents increased as the sample concentration increased. The TPC increased after fermentation from 27.19 (RIESL) and 27.92 (SIESL) to 33.73 (FIESL) GAE mg/g (Table 3). Similarly, the TFC increased by 11.68 (RIESL) and 12.25 (SIESL) to 13.93 (FIESL) RE mg/g (Table 3). Both TPC and TFC exhibited a similar trend depending on the processing method.

Phenolic and flavonoid compounds are abundantly present in plants and can be generated by the influence of light during plant growth and by various stress conditions as part of the plant’s defense mechanisms [40]. In this study, changes in TPC and TFC were observed after sterilization and lactic acid fermentation. The high temperature in sterilization led to a decrease in the phenolic and flavonoid compounds. Xu and Chang [41] reported similar findings where thermal processing resulted in a decrease in TPC and TFC in yellow and black soybeans. However, an increase was observed during lactic acid fermentation, consistent with the findings of various studies indicating increases in TPC and TFC following lactic acid fermentation [2,22,25]. This increase can be attributed to the hydrolytic enzymes produced by LAB, which degrade compounds, such as gallic and vanillic acids, bound to plant cell walls, leading to their release. Khan et al. [22] reported similar observations of dried longan lactic acid fermentation, where the bound phenolic compounds decreased but the free phenolic compounds increased. These phenolic and flavonoid compounds, thus generated, can influence the antioxidant and digestive enzyme inhibitory activities based on the structural characteristics of each compound.

#### 3.3.6. Antioxidant Activities of IESL According to the Processing Stage

Changes in the antioxidant activities of IESL according to the different processing methods are shown in Table 3. In the measured DPPH radical scavenging activity, FIESL showed the highest activity, with IC_50_ values of 0.170, 0.218, and 0.157 mg/mL for RIESL, SIESL, and FIESL, respectively (Table 3). In addition, ABTS radical scavenging activity showed an increase in the order of SIESL < RIESL < FIESL. FIESL showed the highest activity in the ABTS assay, with IC_50_ values of 0.079, 0.088, and 0.074 mg/mL for RIESLs, SIESL, and FIESL, respectively (Table 3). However, the DPPH and ABTS radical scavenging activities of FIESL were not significantly different from those of RIESL and SIESL.

Reactive oxygen species (ROS) are compounds that possess unpaired electrons, rendering them highly unstable and capable of engaging in chemical reactions with other molecules. They can be generated using physiological processes, such as cellular respiration or external stress factors. Although ROS are essential in cell signaling, excessive ROS generation can promote cellular damage and aging [42]. Antioxidants inhibit the action of ROS by scavenging them, thus preventing their attack on other cells. These antioxidant activities are often facilitated by compounds such as phenolic and flavonoid compounds, which possess hydroxyl groups [43]. Therefore, studies have shown that the increase in TPC and TFC correlates with enhanced antioxidant activity [24,38]. Also, in the present study, FIESL were observed to have superior antioxidant activity compared with SIESL. This can be explained by the structural changes in the phenolic and flavonoid compounds, similar to the bioconversion of isoflavones. In the case of isoflavones, conversion from the glycoside to the aglycone type increases the number of hydroxyl groups. Therefore, the increased active hydroxyl groups may have contributed to the enhanced antioxidant activity. In addition, the resonance stability of the aromatic rings can favorably influence the antioxidant activity [44]. FIESL, with their increased contents of phenolic and flavonoid compounds and active hydroxyl groups because of lactic acid fermentation, can be utilized as materials with superior antioxidant activity.

#### 3.3.7. Digestive Enzyme Activities of IESL According to the Processing Stage

Changes in the digestive enzyme inhibitory activities of IESL according to different processing methods are shown in Table 3. The digestive enzyme inhibitory activities of IESL were measured at different concentrations and the IC_50_ values were calculated. As regards α-glucosidase inhibitory activity, FIESL exhibited the highest activity, with IC_50_ values of 3.195, 3.598, and 2.848 mg/mL for RIESL, SIESL, and FIESL, respectively (Table 3). This finding indicates that FIESL show relatively high α-glucosidase inhibitory activity, even at low concentrations. In terms of the pancreatic lipase inhibitory activity, FIESLs exhibited the highest activity in terms of the inhibition of pancreatic lipase, with IC_50_ values of 0.079, 0.088, and 0.074 mg/mL for RIESL, SIESL, and FIESL, respectively (Table 3). This indicates that FIESL demonstrate relatively superior activity in the inhibition of pancreatic lipase.

When food is ingested, digestive enzymes are present in the body. Among them, α-glucosidase acts in the final stage of carbohydrate digestion in the intestinal mucosa, hydrolyzing carbohydrates into monosaccharides. Pancreatic lipase is a fat-digesting enzyme that aids in the absorption of dietary-neutral fats [2]. The inhibition of these digestive enzymes plays an important role in the management of type 2 diabetes and antiobesity activities [45]. Drug therapy and the use of synthetic compounds can lead to side effects, such as gastrointestinal infections and appetite loss; therefore, the consumption of fermented foods is recommended [46]. Recently, Lee et al. [47] confirmed the antiobesity effects of fermented isoflavone-enriched soybean leaves, including weight loss, reduced fat accumulation, and decreased blood glucose and leptin levels. These findings indicate the potential of promising functional foods to prevent and treat obesity by inhibiting digestive enzymes and modulating the gut microbiota. In this study, FIESL fermentation also showed an increase in the inhibitory activities of the digestive enzyme. The decrease during sterilization and subsequent increase during lactic acid fermentation are related to the changes in the contents of phenolic and flavonoid compounds. The structure of phenolic compounds varies depending on the type and the inhibition of digestive enzymes can occur through irreversible inhibition or reversible inhibition (competitive and noncompetitive) by various compounds [18,48,49]. Various studies have reported an increase in the content of phenolic and flavonoid compounds and an increase in digestive enzyme inhibitory activities through lactic acid fermentation [14,25,38]. Therefore, in terms of digestive enzyme inhibitory activities, FIESL exhibit superior activity compared with other processing methods, indicating it may be a beneficial approach for antidiabetic and antiobesity purposes.

#### 3.3.8. DNA Damage Protecting Activity of IESL According to the Processing Stage

**Figure 9:**
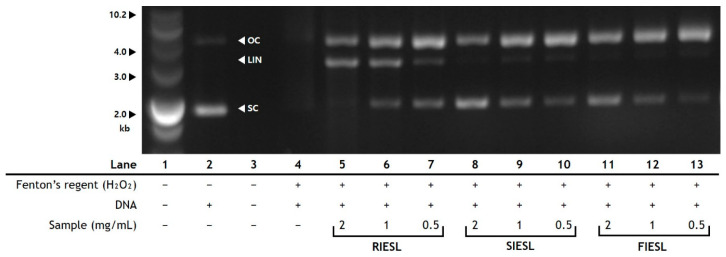
DNA damage protecting activity of IESLs fermented by optimal conditions with lactic acid bacteria strains LAB47 and WCP02. RIESLs, raw isoflavone-enriched soybean leaves; SIESLs, steam isoflavone-enriched soybean leaves; and FIESLs, fermented isoflavone-enriched soybean leaves. Lane 1, size mark; Lane 2, pBluescript SK(+) vector only; Lane 3, blank; Lane 4, pBluescript SK(+) vector with Fenton’s reagent (H_2_O_2_); Lanes 5 to 13, pBluescript SK(+) vector with Fenton’s reagent (H_2_O_2_) and IESL concentrates (5, RIESL 2 mg/mL; 6, RIESL 1 mg/mL; 7, RIESL 0.5 mg/mL; 8, SIESL 2 mg/mL; 9, SIESL 1 mg/mL; 10, SIESL 0.5 mg/mL; 11, FIESL 2 mg/mL; 12, FIESL 1 mg/mL; 13, FIESL 0.5 mg/mL). OC, open circular; LIN, linear; and SC, supercoiled.

To obtain more information about the antioxidant activity of IESL depending on the processing method, the DNA damage protection activity was evaluated (Figure 9). In Lane 2, when only plasmid DNA was loaded, bands in the supercoiled (SC) form were observed; however, bands in the open circular (OC) and linear (LIN) forms were not observed. In Lane 4, the addition of Fenton’s reagent caused DNA damage; as a result, no bands were observed. Lanes 5–13 were loaded with DNA after adding RIESL, SIESL, and FIESL concentrates to Fenton’s reagent at concentrations of 2, 1, and 0.5 mg/mL, respectively. For RIESL, bands in the OC, LIN, and SC forms were observed. As the concentration increased, the bands in the OC and SC forms became fainter, whereas the band in the LIN form became clearer. In contrast, for SIESL and FIESL, as the concentration increased, the bands in the OC form became slightly fainter, whereas the bands in the SC form became clearer. In particular, bands in the LIN form were not observed.

In the RIESL concentrate, an increase in the concentration led to partial DNA damage due to hydroxyl radicals generated by Fenton’s reaction, resulting in the transformation of the band from the SC form to the LIN form. In contrast, in the SIESL and FIESL concentrates, an increase in the concentration resulted in a clearer SC band, indicating the protection of DNA from attack by hydroxyl radicals. Hydroxyl radicals can be neutralized by antioxidants. Therefore, the antioxidant activity of IESL, depending on the processing method, can protect the DNA. Through the study on the antioxidant activity and DNA protection of *Vernonia amygdalina* leaf extracts by Wang et al. [50], the association between the antioxidant activity and DNA protection was confirmed. Although RIESL showed higher TFC, TPC, and antioxidant activity than SIESL, they did not exhibit the same level of DNA protection, as evidenced by the presence of LIN bands. This phenomenon may be attributed to the lack of sterilization. Further studies are needed in this area. Therefore, the FIESL concentrate, which demonstrated DNA protection effects, may have potential effects of preventing cell damage and related diseases caused by ROS.

## 4. Conclusions

In this study, the fermentation characteristics of the LAB isolated from kimchi were examined and the fermentation conditions for IESL were optimized. Excellent GABA production and isoflavone bioconversion were observed under the fermentation conditions of LAB47:WCP02 at a mixing 1:1 ratio for 72 h. Subsequently, IESL were divided into raw, steamed, and fermented materials to analyze changes in physiologically active substances and metabolites. Significant changes were observed in each processing stage. In particular, FIESL exhibited superior isoflavone bioconversion compared with other stages, along with high antioxidant activities, digestive enzyme inhibitory activities, and DNA protective effects. This finding indicates that fermentation using LAB, with excellent fermentation performance, is crucial in enhancing the utility of IESL. This approach can contribute to the potential utilization of soybean leaves in the food industry and this study provides essential baseline data for the development of highly nutritious functional foods.

## Figures and Tables

**Table 1:** Survival capability of 13 lactic acid bacteria strains isolated and selected from kimchi under artificial gastric acidic conditions.

Isolates	Survival Rate (%) ^1^					
	Incubation Time/pH Condition					
	2 h/pH 2.0	4 h/pH 2.0	2 h/pH 2.5	4 h/pH 2.5	2 h/pH 3.0	4 h/pH 3.0
LAB02	nd ^2^	nd	nd	nd	62.03 ± 3.12 ^f^	26.96 ± 0.43 ^cd^
LAB07	24.72 ± 1.06 ^b^	6.26 ± 0.12 ^b^	64.05 ± 2.50 ^a^	26.39 ± 0.99 ^ab^	89.60 ± 0.79 ^b^	49.55 ± 2.01 ^ab^
LAB10	nd	nd	0.12 ± 0.01 ^d^	nd	3.00 ± 0.04 ^j^	nd
LAB11	29.05 ± 0.60 ^a^	9.25 ± 0.21 ^a^	63.32 ± 1.32 ^a^	28.57 ± 0.94 ^a^	95.94 ± 1.21 ^a^	51.96 ± 1.89 ^a^
LAB12	nd	nd	0.22 ± 0.01 ^d^	nd	4.10 ± 0.26 ^ij^	nd
LAB20	nd	nd	0.60 ± 0.03 ^d^	nd	2.31 ± 0.07 ^j^	nd
LAB21	11.88 ± 0.11 ^d^	nd	46.34 ± 1.20 ^c^	7.36 ± 0.24 ^d^	69.73 ± 0.68 ^e^	25.89 ± 0.73 ^d^
LAB22	nd	nd	0.10 ± 0.00 ^d^	nd	16.79 ± 0.33 ^h^	2.67 ± 0.06 ^e^
LAB39	nd	nd	0.84 ± 0.04 ^d^	nd	71.56 ± 3.14 ^de^	30.66 ± 1.37 ^c^
LAB44	nd	nd	0.18 ± 0.01 ^d^	nd	50.00 ± 2.40 ^g^	24.00 ± 0.39 ^d^
LAB47	20.63 ± 0.41 ^c^	4.61 ± 0.20 ^c^	62.22 ± 2.96 ^a^	24.75 ± 0.52 ^b^	82.43 ± 2.57 ^c^	46.02 ± 0.77 ^b^
LAB48	nd	nd	1.59 ± 0.05 ^d^	nd	9.95 ± 0.13 ^i^	nd
LAB49	11.79 ± 0.38 ^d^	nd	54.85 ± 2.03 ^b^	19.16 ± 0.90 ^c^	77.39 ± 1.89 ^cd^	31.01 ± 1.59 ^c^

^1^ Values are presented as mean ± standard deviation for resistance in MRS broth (adjusted pH using HCl and NaOH) supplemented with 1.0% pepsin (*n* = 3). Different superscript letters indicate significant differences relating to the fermented lactic acid bacterial strains, as determined by Tukey’s test (*p* < 0.05). ^2^ nd: not detected.

**Table 2:** Change to the physicochemical properties of isoflavone-enriched soybean leaves during food processing stages.

Index ^1^	Food Processing Stages ^2^		
	RIESL	SIESL	FIESL
pH	6.19 ± 0.01 ^b^	6.17 ± 0.02 ^b^	6.25 ± 0.02 ^a^
Acidity (%, as lactic acid)	2.07 ± 0.01 ^b^	2.07 ± 0.01 ^b^	2.16 ± 0.01 ^a^
Viable cell numbers (log cfu/g)	nm ^3^	nm	8.95± 0.08 ^a^

^1^ All values are presented as the mean ± SD of pentaplicate determinations and different small letters correspond to the significant differences relating to the food processing stages using Tukey’s multiple test (*p* < 0.05). ^2^ Food processing stages: RIESL, raw isoflavone-enriched soybean leaves; SIESL, steamed isoflavone-enriched soybean leaves; and FIESL, fermented isoflavone-enriched soybean leaves. ^3^ nm: not measured.

**Table 3:** Change in total phenolic and total flavonoid contents, antioxidant activities, and digestive enzyme inhibitory activities of isoflavone-enriched soybean leaves during food processing stages.

Index ^1^	Food Processing Stages ^2^		
	RIESL	SIESL	FIESL
Total phenolic contents (GAE mg/g)	27.19 ± 0.04 ^b^	27.92 ± 0.08 ^b^	33.73 ± 0.13 ^a^
Total flavonoid contents (RE mg/g)	11.68 ± 0.04 ^b^	12.25 ± 0.01 ^ab^	13.93 ± 0.03 ^a^
DPPH radical scavenging IC_50_ (mg/mL)	0.17 ± 0.01 ^a^	0.21 ± 0.00 ^b^	0.16 ± 0.01 ^a^
ABTS radical scavenging IC_50_ (mg/mL)	0.08 ± 0.00 ^b^	0.09 ± 0.00 ^a^	0.07 ± 0.00 ^b^
Glucosidase inhibition IC_50_ (mg/mL)	3.19 ± 0.09 ^b^	3.60 ± 0.11 ^a^	2.85 ± 0.08 ^c^
Pancreatic-lipase inhibition IC_50_ (mg/mL)	5.92 ± 0.19 ^b^	6.58 ± 0.17 ^a^	4.38 ± 0.11 ^c^

^1^ All values are presented as the mean ± SD of pentaplicate determinations and different small letters correspond to the significant differences relating to the food processing stages using Tukey’s multiple test (*p* < 0.05). ^2^ Food processing stages: RIESL, raw isoflavone-enriched soybean leaves; SIESL, steamed isoflavone-enriched soybean leaves; and FIESL, fermented isoflavone-enriched soybean leaves.

## Data Availability

The original contributions presented in this study are included in the article/Supplementary Material and further inquiries can be directed to the corresponding authors.

## Supplementary Materials

The following supporting information can be downloaded at: https://www.mdpi.com/article/10.3390/foods14061008/s1, Figure S1: Comparison of β-glycosidase activity and isoflavone ratio in IESL fermentation using different lactic acid bacteria; Figure S2: Comparison of thin-layer chromatography profile in IESL fermentation using different lactic acid bacteria; Figure S3. 16S rRNA sequence of LAB47; Figure S4: Chromatograms of free amino acids of IESL at various processing stages fermented using optimal conditions with lactic acid bacteria strains isolated and selected from *kimchi*; Table S1: Comparison of isoflavone contents in the fermented isoflavone-enriched soybean leaves with different lactic acid bacteria; Table S2: Comparison of isoflavone contents in the fermented isoflavone-enriched soybean leaves with single and mixed strains of LAB47 and WCP02; Table S3: Comparison of isoflavone contents in the fermented isoflavone-enriched soybean leaves with the mixing ratio of LAB47 and WCP02 strains; Table S4: Change in isoflavone contents during fermentation of isoflavone-enriched soybean leaves by the cocktail of LAB47 and WCP02 strains; Table S5: Change in fatty acid contents of isoflavone-enriched soybean leaves during food processing stages; Table S6: Change in free amino acid contents of isoflavone-enriched soybean leaves during food processing stages.

## Abbreviations

The following abbreviations are used in this manuscript:

IESLs	Isoflavone-enriched soybean leaves
RIESLs	Raw isoflavone-enriched soybean leaves
SIESLs	Steamed isoflavone-enriched soybean leaves
FIESLs	Fermented isoflavone-enriched soybean leaves
