# Supplementary material for: Enhancing the Phytochemicals and Antioxidant Abilities of Isoflavone-Enriched Soybean Leaves Through Inoculation with Lacticaseibacillus paracasei LAB47 and Levilactobacillus brevis WCP02
Source: Foods. 2025 Mar 16;14(6):1008. doi: 10.3390/foods14061008 (PMC11941328; doi:10.3390/foods14061008)
Supplement: Supplementary file 1 [file foods-14-01008-s001.zip › foods-3494190-supplementary.pdf]

Supplementary materials

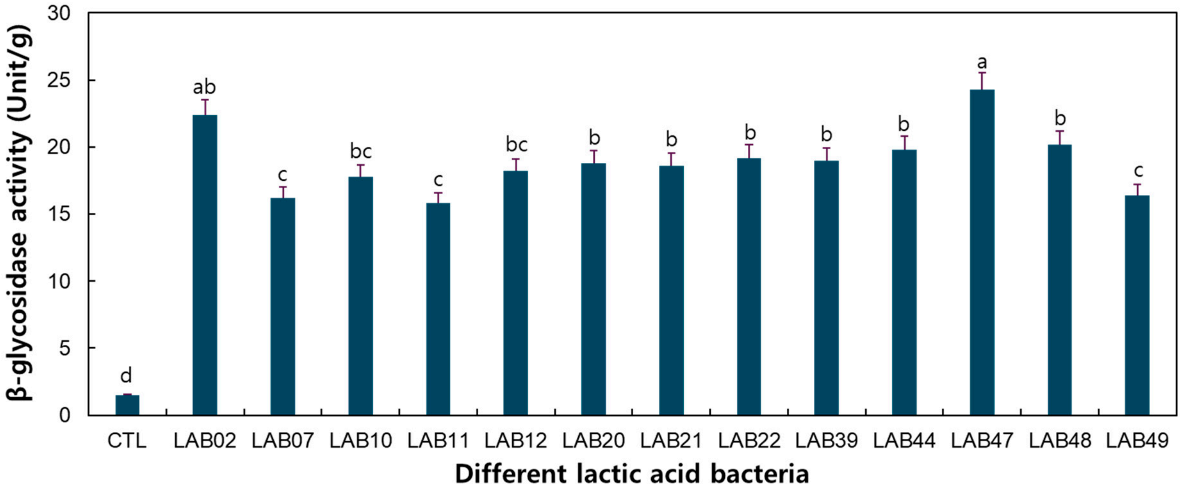

Figure S1. Comparison of β-glycosidase activity and isoflavone ratio in IESL fermentation using different lactic acid bacteria. The isoflavone-enriched soybean leaves were fermented at 30 °C for 120 h using 13 isolated lactic acid bacterial strains. All values are presented as the mean ± SD of pentaplicate determinations, and different small letters correspond to the significant differences relating to the fermented lactic acid bacterial strains using tukey's multiple test ($p < 0.05$).

Table S1. Comparison of isoflavone contents in the fermented isoflavone-enriched soybean leaves with different lactic acid bacteria.

Contents (μg/g)[1]	Fermentation by different LAB strains[2]						
	CTL	LAB02	LAB07	LAB10	LAB11	LAB12	LAB20
**Glycosides**							
Daidzin	2927.57 ± 97.90^{a}	239.60 ± 12.99^{h}	1855.23 ± 23.32^{c}	1644.20 ± 30.09^{d}	2404.82 ± 27.23^{b}	563.10 ± 21.26^{fg}	1078.41 ± 12.80^{e}
Glycitin	691.14 ± 5.40^{a}	232.50 ± 6.48^{f}	560.80 ± 26.03^{b}	571.99 ± 16.51^{b}	582.83 ± 26.32^{b}	438.01 ± 5.62^{d}	532.65 ± 8.59^{bc}
Genistin	2294.20 ± 69.34^{a}	363.16 ± 23.38^{i}	1753.09 ± 60.22^{c}	1703.52 ± 64.11^{c}	1948.17 ± 16.92^{b}	1075.22 ± 13.45^{fg}	1384.07 ± 62.55^{d}
Ononin	78.00 ± 0.89^{c}	97.40 ± 2.28^{a}	67.41 ± 1.57^{f}	48.99 ± 2.13^{g}	70.81 ± 0.87^{def}	48.98 ± 1.41^{g}	52.23 ± 1.77^{g}
Total	5990.91	932.66	4236.53	3968.70	5006.63	2125.31	3047.36
**Malonylglycosides**							
Daidzin	132.49 ± 4.64^{de}	175.24 ± 2.81^{b}	132.83 ± 4.03^{de}	109.38 ± 2.66^{f}	150.42 ± 4.10^{c}	103.77 ± 3.27^{fg}	91.97 ± 2.19^{g}
Glycitin	86.45 ± 1.14^{bc}	66.61 ± 2.80^{ef}	75.17 ± 2.57^{cde}	71.57 ± 2.21^{de}	75.90 ± 2.35^{cde}	65.75 ± 3.33^{efg}	91.42 ± 5.59^{b}
Genistin	279.16 ± 4.91^{a}	181.92 ± 4.05^{c}	207.56 ± 2.44^{b}	157.15 ± 0.21^{def}	206.80 ± 6.57^{b}	134.90 ± 4.43^{gh}	164.44 ± 6.15^{cde}
Total	498.10	423.77	415.56	338.10	433.12	304.42	347.83
**Aglycones**							
Daidzein	381.86 ± 14.88^{h}	1812.40 ± 80.67^{ab}	966.42 ± 18.25^{ef}	1116.67 ± 63.40^{e}	642.28 ± 12.79^{g}	1567.60 ± 102.40^{cd}	1424.53 ± 46.72^{d}
Glycitein	50.65 ± 1.06^{f}	187.78 ± 3.30^{b}	64.42 ± 2.37^{ef}	71.71 ± 1.89^{e}	50.43 ± 1.96^{f}	108.57 ± 6.85^{d}	104.99 ± 4.85^{d}
Genistein	109.80 ± 3.34^{i}	740.06 ± 17.25^{b}	276.86 ± 10.34^{g}	299.51 ± 4.34^{g}	205.57 ± 6.55^{h}	467.23 ± 10.11^{ef}	419.38 ± 19.81^{f}
Formononetin	24.98 ± 0.74^{f}	69.68 ± 3.94^{c}	49.04 ± 1.28^{e}	60.41 ± 0.45^{d}	44.58 ± 1.51^{e}	76.85 ± 3.00^{ab}	77.01 ± 0.93^{ab}
Total	567.29	2809.92	1356.74	1548.30	942.86	2220.25	2025.91
**Total isoflavones**	**7056.30**	**4166.35**	**6008.83**	**5855.10**	**6382.61**	**4649.98**	**5421.10**

[1]All values are presented as the mean ± SD of pentaplicate determinations, and different small letters correspond to the significant differences relating to the fermented lactic acid bacterial strains using tukey's multiple test ($p < 0.05$).

[2]Fermentation conditions: The isoflavone-enriched soybean leaves were fermented at 30 °C for 120 hr using 13 isolated lactic acid bacterial strains.

Table S1. Continued

Contents (μg/g)[1]	Fermentation by different LAB strains[2]						
	LAB21	LAB22	LAB39	LAB44	LAB47	LAB48	LAB49
**Glycosides**							
Daidzin	990.70 ± 29.87^{e}	572.43 ± 5.39^{fg}	658.52 ± 19.58^{f}	356.09 ± 2.99^{h}	96.04 ± 1.82^{i}	521.93 ± 5.97^{g}	1983.06 ± 67.21^{c}
Glycitin	455.74 ± 15.92^{d}	422.46 ± 14.57^{d}	476.31 ± 18.71^{cd}	300.56 ± 9.79^{e}	177.96 ± 5.82^{f}	460.64 ± 27.61^{d}	531.19 ± 3.37^{bc}
Genistin	1285.60 ± 23.39^{de}	1099.69 ± 30.63^{fg}	1151.08 ± 15.21^{ef}	732.86 ± 7.35^{h}	39.97 ± 1.94^{j}	1003.73 ± 29.33^{g}	1737.64 ± 35.53^{c}
Ononin	67.71 ± 1.64^{ef}	75.52 ± 0.44^{cd}	73.91 ± 2.96^{cdef}	74.12 ± 2.60^{cde}	85.43 ± 2.77^{b}	52.57 ± 0.71^{g}	76.20 ± 0.36^{cd}
Total	2799.75	2170.10	2359.82	1463.63	399.40	2038.87	4328.09
**Malonylglycosides**							
Daidzin	203.78 ± 4.10^{a}	171.35 ± 9.64^{b}	119.70 ± 5.81^{ef}	183.88 ± 5.24^{b}	147.40 ± 1.50^{cd}	112.64 ± 3.57^{f}	136.27 ± 2.08^{cd}
Glycitin	122.96 ± 2.48^{a}	60.05 ± 1.80^{fg}	69.31 ± 4.21^{def}	55.27 ± 1.79^{g}	73.03 ± 2.15^{de}	68.44 ± 2.09^{def}	78.56 ± 5.07^{cd}
Genistin	140.43 ± 2.63^{fgh}	172.28 ± 8.28^{cd}	178.72 ± 3.81^{c}	150.08 ± 8.09^{efg}	173.14 ± 2.66^{cd}	124.01 ± 2.39^{h}	178.73 ± 3.96^{c}
Total	467.17	403.68	367.73	389.23	393.57	305.09	393.56
**Aglycones**							
Daidzein	1444.44 ± 91.28^{d}	1625.62 ± 44.99^{bcd}	1570.10 ± 34.18^{cd}	1769.95 ± 59.32^{bc}	1996.68 ± 63.00^{a}	1700.90 ± 22.57^{bc}	854.20 ± 27.86^{f}
Glycitein	103.86 ± 2.27^{d}	105.70 ± 3.13^{d}	116.57 ± 2.39^{d}	152.37 ± 6.43^{c}	255.72 ± 6.19^{a}	141.06 ± 4.26^{c}	65.97 ± 1.26^{e}
Genistein	451.25 ± 18.93^{ef}	486.65 ± 27.85^{de}	472.86 ± 29.39^{ef}	614.30 ± 12.79^{c}	870.23 ± 26.58^{a}	545.01 ± 13.02^{d}	256.89 ± 10.11^{gh}
Formononetin	61.16 ± 1.91^{d}	69.19 ± 1.30^{c}	71.48 ± 2.63^{bc}	73.83 ± 1.40^{abc}	79.41 ± 0.19^{a}	73.21 ± 1.83^{abc}	45.47 ± 0.37^{e}
Total	2060.71	2287.16	2231.01	2610.45	3202.04	2460.18	1222.53
**Total isoflavones**	**5327.63**	**4860.94**	**4958.56**	**4463.31**	**3995.01**	**4804.14**	**5944.18**

[1]All values are presented as the mean ± SD of pentaplicate determinations and different small letters correspond to the significant differences relating to the fermented lactic acid bacterial strains using tukey's multiple test ($p < 0.05$).

[2]Fermentation conditions: The isoflavone-enriched soybean leaves were fermented at 30 °C for 120 hr using 13 isolated lactic acid bacterial strains.

Figure S2

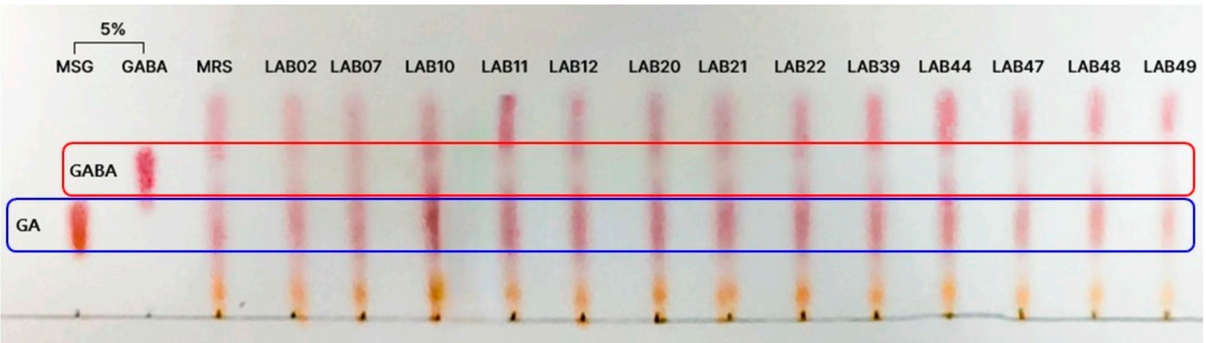

Figure S2. Comparison of thin-layer chromatography profile in IESL fermentation using different lactic acid bacteria. GA, glutamic acid; GABA, γ-aminobutyric acid.

Figure S3

16S RNA sequences of LAB47

```
   1 GTTCTCGTTG ATGATCGGTG CTTGCACCGA GATTCAACAT GGAACGAGTG GCGGACGGGT
  61 GAGTAACACG TGGGTAACCT GCCCTTAAGT GGGGGATAAC ATTTGGAAAC AGATGCTAAT
 121 ACCGCATAGA TCCAAGAACC GCATGGTTCT TGGCTGAAAG ATGGCGTAAG CTATCGCTTT
 181 TGGATGGACC CGCGGCGTAT TAGCTAGTTG GTGAGGTAAN GGCTCACCAA GGCGATGATA
 241 CGTAGCCGAA CTGAGAGGTT GATCGGCCAC ATTGGGACTG AGACACGGCC CAAACTCCTA
 301 CGGGAGGCAG CAGTAGGGAA TCTTCCACAA TGGACGCAAG TCTGATGGAG CAACGCCGCG
 361 TGAGTGAAGA AGGCTTTCGG GTCGTAAAAC TCTGTTGTTG GAGAAGAATG GTCGGCAGAG
 421 TAACTGTTGC CGGCGTGACG GTATCCAACC AGAAAGCCAC GGCTAACTAC GTGCCAGCAG
 481 CCGCGGTAAT ACGTAGGTGG CAAGCGTTAT CCGGATTTAT TGGGCGTAAA GCGAGCGCAG
 541 GCGGTTTTTT AAGTCTGATG TGAAAGCCCT CGGCTTAACC GAGGAAGCGC ATCGGAAACT
 601 GGGAAACTTG AGTGCAGAAG AGGACAGTGG AACTCCATGT GTAGCGGTGA AATGCGTAGA
 661 TATATGGAAG AACACCAGTG GCGAAGGCGG CTGTCTGGTC TGTAACTGAC GCTGAGGCTC
 721 GAAAGCATGG GTAGCGAACA GGATTAGATA CCCTGGTAGT CCATGCCGTA AACGATGAAT
 781 GCTAGGTGTT GGAGGGTTTC CGCCCTTCAG TGCCGCAGCT AACGCATTAA GCATTCCGCC
 841 TGGGGAGTAC GACCGCAAGG TTGAAACTCA AAGGAATTGA CGGNGGCCCG CACAAGCGGT
 901 GGAGCATGTG GTTTAATTCG AAGCAACGCG AAGAACCTTA CCAGGTCTTG ACATCTTTTG
 961 ATCACCTGAG AGATCAGGTT TCCCCTTCGG GGGCAAAATG ACAGGTGGTG CATGGTTGTC
1021 GTCAGCTCGT GTCGTGAGAT GTTGGGTTAA GTCCCGCAAC GAGCGCAACC CTTATGACTA
1081 GTTGCCAGCA TTTAGTTGGG CACTCTAGTA AGACTGCCGG TGACAAACCG GAGGAAGGTG
1141 GGGATGACGT CAAATCATCA TGCCCCTTAT GACCTGGGCT ACACACGTGC TACAATGGAT
1201 GGTACAACGA GTTGCGAGAC CGCGAGGTCA AGCTAATCTC TTAAAGCCAT TCTCAGTTCG
1261 GACTGTAGGC TGCAACTCGC CTACACGAAG TCGGAATCGC TAGTAATCGC GGATCAGCAC
1321 GCCGCGGTGA ATACGTTCCC GGGCCTTGTA CACACCGCCC GTCACACCAT GAGAGTTTGT
1381 AACACCCGAA GCCGGTGGCG TAACCCT
```

Figure S3. 16S rRNA sequence of LAB47.

Table S2. Comparison of isoflavone contents in the fermented isoflavone-enriched soybean leaves with single and mixed strains of LAB47 and WCP02.

**Contents (μg/g)[1]**	**Fermentation by single and mixed strains of LAB47 and WCP02[2]**			
	**CTL**	**LAB47**	**WCP02**	**LAB47 + WCP02**
		**Glycosides**		
Daidzin	4834.34 ± 110.73^{a}	147.01 ± 3.07^{c}	2324.98 ± 85.99^{b}	116.05 ± 2.32^{c}
Glycitin	1210.29 ± 44.16^{a}	254.77 ± 9.43^{c}	813.91 ± 26.23^{b}	245.07 ± 8.64^{c}
Genistin	3852.51 ± 121.85^{a}	63.76 ± 1.29^{c}	527.97 ± 33.96^{b}	65.63 ± 2.81^{c}
Ononin	190.49 ± 3.59^{b}	222.98 ± 8.02^{a}	217.85 ± 3.27^{a}	167.42 ± 4.59^{c}
Total	10087.63	688.52	3884.71	594.17
		**Malonylglycosides**		
Daidzin	306.12 ± 4.92^{a}	254.95 ± 9.67^{b}	285.68 ± 10.67^{a}	249.69 ± 4.77^{b}
Glycitin	122.69 ± 4.29^{b}	152.71 ± 4.86^{a}	131.48 ± 2.26^{b}	127.59 ± 2.64^{b}
Genistin	473.87 ± 14.35^{a}	310.20 ± 15.67^{b}	316.52 ± 8.75^{b}	307.73 ± 7.41^{b}
Total	902.68	717.86	733.68	685.01
		**Aglycones**		
Daidzein	677.71 ± 24.10^{c}	3331.76 ± 120.78^{a}	2187.99 ± 67.04^{b}	3310.77 ± 119.45^{a}
Glycitein	99.22 ± 2.69^{d}	442.56 ± 12.33^{a}	265.38 ± 9.63^{c}	393.50 ± 10.90^{b}
Genistein	185.43 ± 4.67^{c}	1412.97 ± 44.90^{a}	1286.90 ± 39.72^{b}	1423.92 ± 45.24^{a}
Formononetin	54.93 ± 1.95^{c}	152.96 ± 7.05^{a}	145.74 ± 5.51^{ab}	135.52 ± 1.88^{b}
Total	1017.29	5340.25	3886.01	5263.71
**Total isoflavones**	**12007.60**	**6746.63**	**8504.40**	**6542.89**

[1]All values are presented as the mean ± SD of pentaplicate determinations and different small letters correspond to the significant differences relating to single and mixed strains of LAB47 and WCP02 using tukey's multiple test ($p < 0.05$).

[2]Fermentation conditions: The IESL was fermented at 30 °C for 120 h with single and mixed strains of LAB47 and WCP02.

Table S3. Comparison of isoflavone contents in the fermented isoflavone-enriched soybean leaves with the mixing ratio of LAB47 and WCP02 strains.

Contents (μg/g)[1]	Fermentation by the mixing ratio of LAB47 and WCP02[2]					
	CTL	1:1	2:1	3:1	1:2	1:3
			**Glycosides**			
Daidzin	2747.84 ± 98.83^a	78.68 ± 2.54^b	79.87 ± 2.00^b	77.25 ± 2.74^b	80.22 ± 2.21^b	87.46 ± 3.11^b
Glycitin	700.24 ± 30.15^a	56.28 ± 2.70^b	96.13 ± 0.45^b	92.17 ± 3.50^b	62.23 ± 2.77^b	60.71 ± 1.44^b
Genistin	2269.72 ± 37.75^a	26.11 ± 0.48^b	27.46 ± 1.47^b	24.59 ± 0.77^b	28.67 ± 1.01^b	28.47 ± 0.84^b
Ononin	87.83 ± 4.36^a	64.38 ± 1.48^b	65.38 ± 2.28^b	61.55 ± 1.98^b	67.40 ± 2.17^b	61.02 ± 2.45^b
Total	5805.64	225.44	268.85	255.55	238.52	237.67
			**Malonylglycosides**			
Daidzin	122.77 ± 4.32^a	129.35 ± 4.45^a	117.49 ± 2.46^a	124.33 ± 4.17^a	131.34 ± 6.16^a	126.87 ± 6.35^a
Glycitin	94.27 ± 2.16^a	55.85 ± 2.68^c	71.37 ± 2.39^b	54.24 ± 3.03^c	58.74 ± 0.68^c	55.50 ± 0.71^c
Genistin	265.96 ± 13.45^a	132.92 ± 4.84^b	131.79 ± 4.30^b	127.03 ± 1.86^b	143.92 ± 3.50^b	122.75 ± 4.23^b
Total	483.01	318.11	320.65	305.59	333.99	305.11
			**Aglycones**			
Daidzein	412.18 ± 19.84^b	1898.77 ± 30.14^a	1940.37 ± 15.39^a	1821.84 ± 118.53^a	1994.60 ± 102.53^a	1952.31 ± 65.68^a
Glycitein	51.46 ± 1.63^d	221.05 ± 2.81^{bc}	230.77 ± 3.80^{ab}	209.75 ± 2.94^c	242.89 ± 3.62^a	224.02 ± 5.96^b
Genistein	102.46 ± 2.10^c	866.25 ± 28.01^{ab}	884.63 ± 22.32^{ab}	829.43 ± 28.37^b	917.33 ± 24.69^a	902.86 ± 15.88^{ab}
Formononetin	24.28 ± 0.43^b	77.24 ± 2.90^a	80.31 ± 0.39^a	77.88 ± 3.63^a	84.45 ± 2.46^a	81.43 ± 1.73^a
Total	590.37	3063.32	3136.07	2938.90	3239.27	3160.63
**Total isoflavones**	**6879.02**	**3606.87**	**3725.57**	**3500.05**	**3811.78**	**3703.41**

[1]All values are presented as the mean ± SD of pentaplicate determination and different small letters correspond to the significant differences relating to the mixing ratio of LAB17 and WCP02 strains using tukey's multiple test ($p < 0.05$).

[2]Fermentation conditions: The IESL was fermented at 30 °C for 120 h with the mixing ratio of LAB17 and WCP02 strains.

Table S4. Change in isoflavone contents during fermentation of isoflavone-enriched soybean leaves by the cocktail of LAB47 and WCP02 strains.

Contents (μg/g)[1]	Fermentation time of LAB47 and WCP02 strains (hr)[2]							
	0	6	12	24	36	48	72	96
**Glycosides**								
Daidzin	4336.33 ± 34.44[b]	4462.24 ± 35.55[a]	1399.63 ± 45.98[c]	119.58 ± 3.00[d]	129.68 ± 4.38[d]	112.56 ± 1.84[d]	128.11 ± 1.98[d]	137.48 ± 2.98[d]
Glycitin	955.00 ± 27.41[a]	786.61 ± 7.91[b]	306.40 ± 9.80[c]	39.19 ± 0.79[d]	24.01 ± 0.56[d]	26.96 ± 0.67[d]	27.41 ± 1.40[d]	29.11 ± 0.35[d]
Genistin	3346.69 ± 111.79[a]	3131.29 ± 138.26[a]	823.47 ± 17.67[b]	68.96 ± 1.76[c]	67.73 ± 3.03[c]	56.94 ± 2.13[c]	59.47 ± 2.80[c]	65.13 ± 1.14[c]
Ononin	105.05 ± 3.50[b]	114.48 ± 4.3[ab]	118.66 ± 3.75[a]	119.40 ± 3.46[a]	116.55 ± 2.38[a]	72.38 ± 1.96[d]	89.86 ± 2.48[c]	93.72 ± 3.16[c]
Total	8743.07	8494.62	2648.16	347.13	337.97	268.84	304.85	325.44
**Malonylglycosides**								
Daidzin	230.05 ± 8.48[a]	230.32 ± 241[a]	179.96 ± 5.68[b]	148.76 ± 6.73[cd]	158.47 ± 3.90[c]	120.09 ± 3.60[e]	137.98 ± 3.68[de]	146.25 ± 8.62[cd]
Glycitin	31.29 ± 0.63[bc]	32.84 ± 1.05[b]	39.48 ± 1.28[a]	39.70 ± 0.49[a]	25.75 ± 0.84[d]	28.72 ± 0.59[c]	22.45 ± 0.36[e]	21.61 ± 1.02[e]
Genistin	316.55 ± 7.19[a]	291.50 ± 8.95[ab]	227.38 ± 11.86[c]	314.09 ± 5.56[a]	276.90 ± 9.29[b]	281.14 ± 2.73[b]	209.32 ± 1.76[cd]	192.25 ± 12.30[d]
Total	577.89	554.66	446.82	502.55	461.12	429.95	369.75	360.11
**Aglycones**								
Daidzein	727.79 ± 4.21[d]	869.76 ± 36.86[d]	2512.03 ± 24.96[c]	3331.70 ± 143.64[ab]	3186.29 ± 190.80[b]	3377.71 ± 125.58[ab]	3149.29 ± 56.29[b]	3535.70 ± 46.47[a]
Glycitein	118.50 ± 4.08[d]	164.35 ± 1.48[d]	368.56 ± 7.99[c]	488.35 ± 13.42[ab]	467.45 ± 31.94[ab]	497.28 ± 8.99[a]	444.77 ± 10.88[b]	487.46 ± 8.73[ab]
Genistein	253.21 ± 12.48[c]	396.10 ± 11.17[c]	1250.97 ± 31.38[b]	1570.38 ± 69.05[a]	1487.16 ± 48.56[a]	1568.87 ± 57.10[a]	1461.49 ± 22.06[a]	1627.88 ± 95.87[a]
Formononetin	56.97 ± 1.59[c]	59.27 ± 2.12[c]	108.22 ± 1.12[b]	142.62 ± 4.32[a]	137.12 ± 6.25[a]	142.13 ± 3.64[a]	132.03 ± 5.55[a]	146.39 ± 6.89[a]
Total	1156.47	1489.48	4239.78	5533.05	5278.02	5585.99	5187.58	5797.43
**Total isoflavones**	**10477.43**	**10538.76**	**7334.76**	**6382.73**	**6077.11**	**6284.78**	**5862.18**	**6482.98**

[1]All values are presented as the mean ± SD of pentaplicate determinations, and different small letters correspond to the significant differences relating to the fermentation times using the cocktail of LAB47 and WCP02 (1:1) strains, as determined by tukey's multiple comparison test ($p < 0.05$).

[2]Fermentation time: The IESL was fermented at 30 °C for 96 h using the cocktail LAB47 and WCP02 strains during fermentation.

Table S5. Change in fatty acid contents of isoflavone-enriched soybean leaves during food processing stages.

Contents (mg/100 g)[1]	Food processing stages[2]		
	RIESL	SIESL	FIESL
**Saturated fatty acids (SFA)**			
Lauric acid (C12:0)	6.20 ± 0.13[b]	4.70 ± 0.09[c]	10.20 ± 0.29[a]
Myristic acid (C14:0)	20.40 ± 0.40[a]	14.10 ± 0.21[b]	21.00 ± 0.48[a]
Palmitic acid (C16:0)	434.50 ± 4.49[b]	410.30 ± 6.54[c]	469.70 ± 10.20[a]
Stearic acid (C18:0)	131.10 ± 1.94[b]	126.20 ± 2.31[c]	140.30 ± 2.32[a]
Arachidic acid (C20:0)	13.80 ± 0.28[b]	13.20 ± 0.31[c]	15.10 ± 0.13[a]
Behenic acid (C22:0)	12.60 ± 0.39[b]	12.30 ± 0.38[b]	14.30 ± 0.34[a]
Lignoceric acid (C24:0)	17.80 ± 0.44[b]	17.00 ± 0.42[b]	20.30 ± 0.50[a]
Total	636.40	597.80	690.90
**Unsaturated fatty acids (USFA)**			
Oleic acid (C18:1c)	54.70 ± 1.17[a]	52.30 ± 0.75[b]	53.30 ± 0.93[ab]
Linoleic acid (C18:2c)	109.00 ± 2.22[b]	111.40 ± 1.57[b]	118.40 ± 2.80[a]
α-linolenic acid (C18:3n3)	127.30 ± 3.53[b]	124.40 ± 2.05[b]	136.10 ± 4.08[a]
Eicosadienoic acid (C20:2)	nd[3]	nd	4.20 ± 0.12
Arachidonic acid (C20:4n6)	11.00 ± 0.18[b]	9.20 ± 0.23[c]	12.50 ± 0.37[a]
Docosadienoic acid (C22:2)	11.80 ± 0.21[b]	12.00 ± 0.13[b]	14.30 ± 0.30[a]
Nervonic acid (C24:1n9)	3.70 ± 0.07[b]	3.80 ± 0.07[b]	4.30 ± 0.10[a]
Total	317.50	313.10	343.10
**Total fatty acids**	**953.90**	**910.90**	**1034.00**

[1]All values are presented as the mean ± SD of pentaplicate determinations and different small letters correspond to the significant differences relating to the food processing stages using tukey's multiple test ($p < 0.05$).
[2]Food processing stages: RIESL, raw isoflavone-enriched soybean leaves; SIESL, steamed isoflavone-enriched soybean leaves; and FIESL, fermented isoflavone-enriched soybean leaves.
[3]nd: not detected.

Table S6. Change in free amino acid contents of isoflavone-enriched soybean leaves during food processing stages.

Contents (mg/100 g)[1]	Food processing by different stages[2]		
	RIESL	SIESL	FIESL
**Non-essential amino acids**			
Phosphoserine	41.36 ± 0.77^{a}	27.95 ± 0.94^{c}	35.58 ± 0.67^{b}
Taurine	12.79 ± 0.34^{a}	10.28 ± 0.24^{b}	9.97 ± 0.27^{b}
Proline	100.70 ± 1.71^{a}	79.65 ± 1.00^{b}	102.82 ± 1.88^{a}
Aspartic acid	132.01 ± 2.08^{b}	98.21 ± 2.62^{c}	141.08 ± 2.42^{a}
Serine	64.02 ± 1.18^{a}	39.82 ± 0.90^{b}	40.93 ± 0.99^{b}
Aspartic acid - NH_2	482.73 ± 6.22^{a}	328.55 ± 5.31^{b}	339.68 ± 8.97^{b}
Glutamic acid	92.01 ± 1.53^{a}	50.57 ± 0.96^{b}	18.21 ± 0.41^{c}
Aminoadipic acid	8.64 ± 0.24^{b}	3.75 ± 0.09^{c}	44.47 ± 0.89^{a}
Glycine	15.08 ± 0.93^{b}	10.25 ± 0.24^{b}	18.02 ± 0.41^{a}
Alanine	101.22 ± 2.51^{a}	67.33 ± 1.79^{c}	75.59 ± 1.71^{b}
α-aminobutyric acid	nd[3]	nd	6.44 ± 0.13
Tyrosine	51.52 ± 1.34^{a}	22.85 ± 0.40^{b}	nd
β-alanine	8.71 ± 0.19^{b}	22.23 ± 0.20^{a}	8.05 ± 0.16^{c}
β-aminoisobutyric acid	2.22 ± 0.04^{b}	nd	2.73 ± 0.09^{a}
γ-aminobutyric acid	91.53 ± 2.06^{b}	61.21 ± 1.33^{c}	101.39 ± 2.56^{a}
Aminoethanol	11.91 ± 0.29^{a}	nd	11.47 ± 0.39^{a}
Ornithine	nd	nd	55.27 ± 1.32
Arginine	131.49 ± 1.80^{a}	82.10 ± 1.63^{b}	nd
Total	1526.67	1021.67	1075.74
**Essential amino acids**			
Threonine	28.43 ± 0.58^{a}	13.62 ± 0.37^{b}	8.17 ± 0.21^{c}
Valine	95.79 ± 1.67^{a}	66.74 ± 1.10^{c}	75.26 ± 2.53^{b}
Methionine	5.37 ± 0.12	nd	nd
Isoleucine	51.14 ± 1.02^{a}	34.47 ± 0.69^{c}	39.64 ± 0.63^{b}
Leucine	47.49 ± 0.68^{a}	20.24 ± 0.48^{c}	34.77 ± 0.67^{b}
Phenylalanine	92.08 ± 2.55^{a}	45.83 ± 1.33^{c}	55.67 ± 1.83^{b}
Lysine	40.35 ± 0.90^{a}	18.80 ± 0.68^{b}	6.42 ± 0.10^{c}
Histidine	12.10 ± 0.19	nd	nd
Total	372.75	199.70	219.93
**Total amino acids**	**1899.42**	**1221.37**	**1295.67**
Urea	178.73 ± 2.64^{a}	116.92 ± 2.68^{b}	64.04 ± 1.19^{c}
Ammonia	24.18 ± 0.25^{b}	15.44 ± 0.42^{c}	25.34 ± 0.59^{a}

[1]All values are presented as the mean ± SD of pentaplicate determinations and different small letters correspond to the significant differences relating to the food processing stages using tukey's multiple test ($p < 0.05$).
[2]Food processing stages: RIESL, raw isoflavone-enriched soybean leaves; SIESL, steamed isoflavone-enriched soybean leaves; and FIESL, fermented isoflavone-enriched soybean leaves.
[3]nd: not detected.

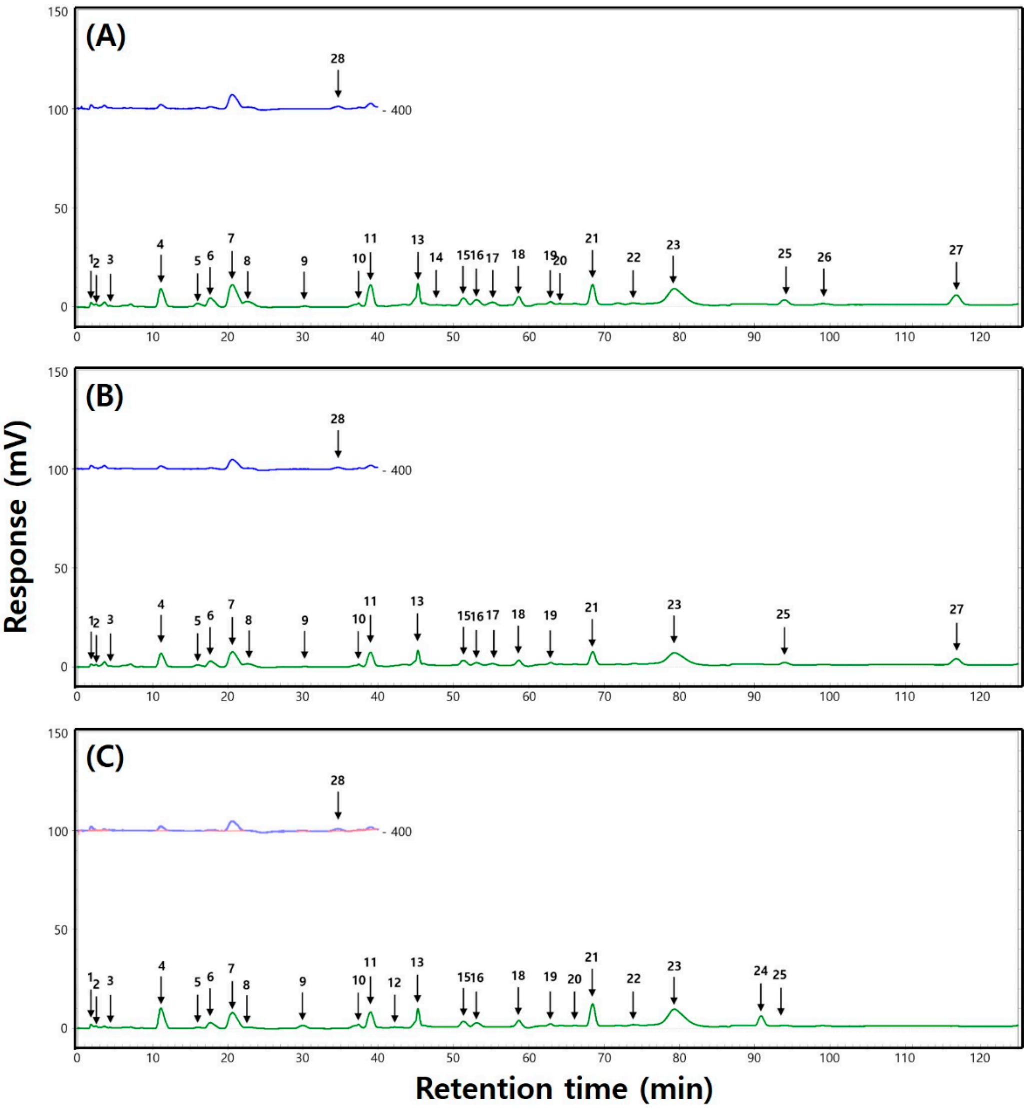

Figure S4. Chromatograms of free amino acids of IESL at various processing stages fermented using optimal conditions with lactic acid bacteria strains isolated and selected from *kimchi*. A, RIESL; B, SIESL; and C, FIESL. RIESL, raw isoflavone-enriched soybean leaves; SIESL, steamed isoflavone-enriched soybean leaves; and FIESL, fermented isoflavone-enriched soybean leaves. Peak 1, phosphoserine; peak 2, taurine; peak 3, urea; peak 4, aspartic acid; peak 5, threonine; peak 6, serine; peak 7, aspartic acid - NH_2; peak 8, glutamic acid; peak 9, aminoadipic acid; peak 10, glycine; peak 11, alanine; peak 12, α-aminobutyric acid; peak 13, valine; peak 14, methionine; peak 15, isoleucine; peak 16, leucine; peak 17, tyrosine; peak 18, phenylalanine; peak 19, β-alanine; peak 20, β-aminoisobutyric acid; peak 21, γ-aminobutyric acid; peak 22, aminoethanol; peak 23, ammonia; peak 24, ornithine; peak 25, lysine; peak 26, histidine; peak 27, arginine; peak 28, proline.
